# Cortical Surround Interactions and Perceptual Salience via Natural Scene Statistics

**DOI:** 10.1371/journal.pcbi.1002405

**Published:** 2012-03-01

**Authors:** Ruben Coen-Cagli, Peter Dayan, Odelia Schwartz

**Affiliations:** 1 Dominick Purpura Department of Neuroscience, Albert Einstein College of Medicine, Bronx, New York, United States of America; 2 Gatsby Computational Neuroscience Unit, University College, London, United Kingdom; 3 Department of Systems and Computational Biology, Albert Einstein College of Medicine, Bronx, New York, United States of America; Indiana University, United States of America

## Abstract

Spatial context in images induces perceptual phenomena associated with salience and modulates the responses of neurons in primary visual cortex (V1). However, the computational and ecological principles underlying contextual effects are incompletely understood. We introduce a model of natural images that includes grouping and segmentation of neighboring features based on their joint statistics, and we interpret the firing rates of V1 neurons as performing optimal recognition in this model. We show that this leads to a substantial generalization of divisive normalization, a computation that is ubiquitous in many neural areas and systems. A main novelty in our model is that the influence of the context on a target stimulus is determined by their degree of statistical dependence. We optimized the parameters of the model on natural image patches, and then simulated neural and perceptual responses on stimuli used in classical experiments. The model reproduces some rich and complex response patterns observed in V1, such as the contrast dependence, orientation tuning and spatial asymmetry of surround suppression, while also allowing for surround facilitation under conditions of weak stimulation. It also mimics the perceptual salience produced by simple displays, and leads to readily testable predictions. Our results provide a principled account of orientation-based contextual modulation in early vision and its sensitivity to the homogeneity and spatial arrangement of inputs, and lends statistical support to the theory that V1 computes visual salience.

## Author Summary

One of the most important and enduring hypotheses about the way that mammalian brains process sensory information is that they are exquisitely attuned to the statistical structure of the natural world. This allows them to come, over the course of development, to represent inputs in a way that reflects the facets of the environment that were responsible. We focus on the case of information about the local orientation of visual input, a basic level feature for which a wealth of phenomenological observations are available to constrain and validate computational models. We suggest a new account which focuses on the statistics of orientations at nearby locations in visual space, and captures data on how such contextual information modulates both the responses of neurons in the primary visual cortex, and the corresponding psychophysical percepts. Our approach thus helps elucidate the computational and ecological principles underlying contextual processing in early vision; provides a number of predictions that are readily testable with existing experimental approaches; and indicates a possible route for examining whether similar computational principles and operations also support higher-level visual functions.

## Introduction

Contextual influences collectively denote a variety of phenomena associated with the way information is integrated and segmented across the visual field. Spatial context strongly modulates the perceptual salience of even simple visual stimuli, as well as influencing cortical responses, as early as in V1 [1]–[12] (for a review, see [13]).

At least two main lines of theoretical inquiry have addressed these influences from different perspectives. First, computational models have related perceptual salience to low-level image features [14]–[18]. Of particular note for us, [14], [19]–[21] proposed that V1 builds a visual saliency map, performing segmentation where the spatial homogeneity of the input breaks down (e.g., at the border between textures). A model of the dynamical, recurrent, interactions among nearby cortical neurons induced by long-range horizontal connections realized this theory, accounting for cortical and perceptual contextual data, including popout, visual search asymmetries, and contour integration [14], [19]–[24]. Second, the hypothesis that sensory processing is optimized to the statistics of the natural environment [25]–[27], has led to successful models of the linear and non-linear properties of V1 receptive fields (RFs) [21], [28]–[35].

**Figure 1:**
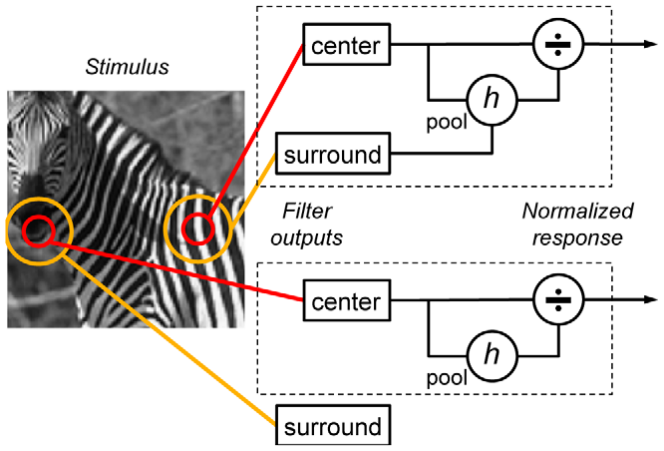
Stimulus-dependent divisive normalization. Top row: cartoon of a divisive normalization model that accounts for surround modulation of V1 responses. In a textured, homogeneous visual stimulus, the center and surround of a V1 neuron's RF (schematically illustrated by red and orange circles, respectively) receive similar inputs. The model pools together the corresponding outputs (computed by oriented linear filters), and combines them (here generically denoted by a function *h*; see Equations 4,6) to generate the signal that divisively normalizes the center output. Bottom row: cartoon of a divisive normalization model that accounts for the absence of surround modulation on heterogeneous visual stimuli (i.e., different features or textures stimulate the center and surround). The model uses only the center outputs to compute the normalization signal (see Equations 5,6).

However, although collectively covering a huge range of computational, psychophysical, and neural data, these two theoretical approaches have not been unified. To this end, we introduce a computational model of the statistical dependencies of neighboring regions in images, rooted in recent advances in computer vision [36]–[39]. This model provides a formal treatment of the idea of statistical homogeneity vs heterogeneity of visual inputs, to which V1 has been proposed to be sensitive [20]. Correct inference in the model involves a novel, generalized, form of divisive surround normalization [30], [40]–[43], that is engaged by stimuli comprising extended single objects (e.g. in Fig. 1 the image patch inside the region with uniform vertical texture), but not by stimuli involving independent visual features (e.g. in Fig. 1 the patch across the zebra and the background).

We focused in particular on the dependencies between orientations across space, optimizing model parameters based on natural scenes. We used the resulting model to simulate neural and perceptual responses to stimuli used in physiological and perceptual experiments to test orientation-based surround modulation. Note that we did not fit the model to experimental data from individual cells or subjects, but rather compared the qualitative behavior of a model trained on ecologically relevant stimuli with general properties of early visual surround modulation.

There is a wealth of experimental results on surround modulation, some classical, and some still subject to debate. We chose to model a set of findings that we intend to be canonical examples of both sorts of results. We included data that have been the subject of previous theoretical treatments, but paid particular attention to phenomena that lie at the boundary between integration and segmentation. Indeed, one claim from our approach is that subtleties at this border might help explain some of the complexities of the experimental findings. We make predictions for regions of the stimulus space that have not yet been fully tested in experiments.

In sum, we show that the statistical principles introduced can account for a range of neural phenomena that demand tuned surround suppression as well as facilitation, and encompass V1 as a salience map [20].

## Materials and Methods

We first illustrate a class of characteristic statistical dependencies across space in natural scenes, and we outline a model of such dependencies. We provide implementation details and model equations, and describe how model parameters were optimized for an ensemble of natural scenes. Further, we explain how the model relates to contextual modulation in the visual cortex. We specifically focus on relating the statistical model of images to V1 neural firing rates, and also illustrate how this constitutes a generalized form of divisive normalization.

### Statistical model of spatial dependencies in scenes

**Figure 2:**
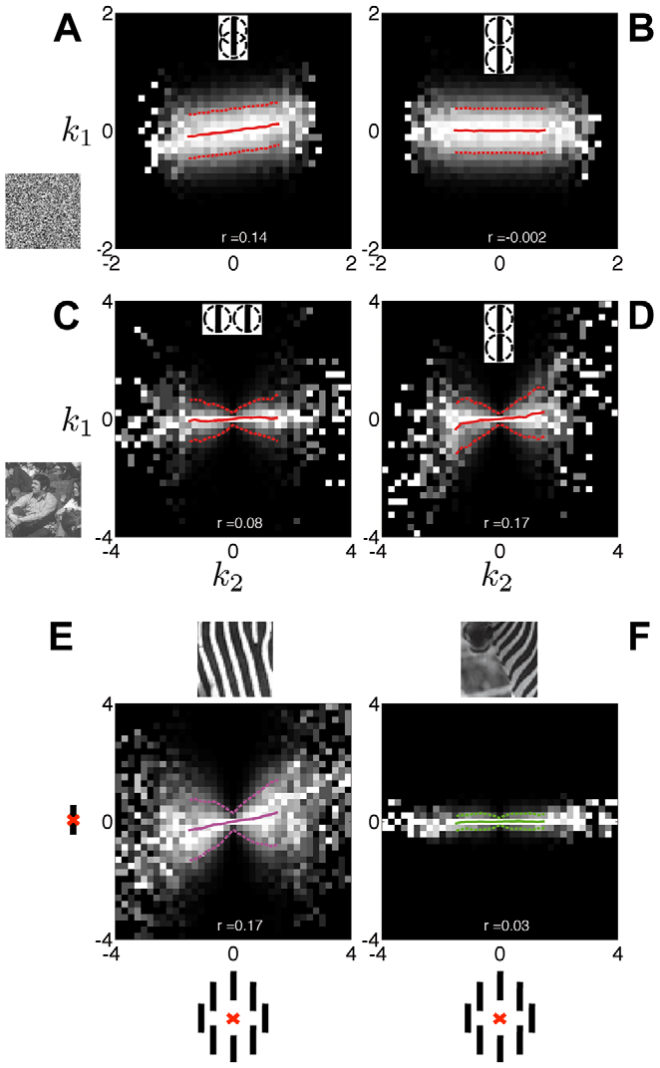
Dependencies amongst oriented filters vary with spatial layout, image set, and across image regions. (**A–D**) Histograms of the outputs of one RF (*k*_1_) given the outputs of the other RF (*k*_2_). We computed these conditional histograms based on 100,000 Gaussian white noise image patches (**A,B**), or 100,000 natural image patches (**C,D**; the images are shown in Fig. S1). Pixel intensity is proportional to probability, larger values correspond to brighter pixels; we rescaled each column independently to fill the range of intensities. Solid and dashed lines denote the mean and standard deviation, respectively, of *k*_1_ for each given value of *k*_2_. We matched the average RMS contrast of noise and natural images; the larger range of RFs responses to natural images reflects the abundance of oriented features that are optimal for the RFs. The insets illustrate the orientation and relative position of the RFs: (**A**) collinear RFs with large overlap (3 pixels separation); (**B,D**) collinear, and (**C**) parallel but not collinear RFs, with minimal overlap (6 pixel separation). The *bowtie* shape in (**C,D**) shows that the variance of *k*_1_ depends on the magnitude of *k*_2_, which is typical of natural images. Further, we report the Pearson correlation coefficient between *k*_1_ and *k*_2_ at the bottom: the stronger linear dependence between collinear filters reflects the predominance of elongated edges and contours in scenes. (**E,F**) Histograms of the outputs of two spatially separated vertical RFs, at center (Y axis) and surround (X axis) locations, averaged across 8 surround locations as illustrated in the axis labels (black bars denote filters, the red cross denotes the center position; surround RFs are 6 pixels away from the center). In (**E**) we included only the subset of the patches in (**C,D**) that were best described by a model with statistically dependent center and vertical surround RFs; whereas in (**F**) we used the patches best described by a model that assumes independence between center and surround RFs (see Materials and Methods for model details). The variance dependence is weaker in (**F**) than (**E**).

We concentrated on the statistical dependencies between V1-like filters (or receptive fields, RFs; see also Text S1) across space in natural images (the images are shown in Fig. S1). We adopted RFs derived from the first level of a steerable pyramid [44]; more details are provided below. For conciseness, we will refer to the projection of a visual stimulus onto an RF as the RF *output*. Fig. 2A–D show the joint conditional histograms of the output of one vertical RF given the output of another vertical RF in a different spatial position. In the case of white noise image patches, outputs are linearly correlated for RFs that overlap in space (Fig. 2A), but not for RFs that are farther apart and so non-overlapping (Fig. 2B). Natural scenes differ from white noise. The characteristic bowtie shape of Fig. 2C,D indicates statistical coordination in the form of a higher-order variance dependency [30]: i.e., the variance of one RF depends on the magnitude of the output of the other. Further, elongated structures, such as edges and contours, cause strong co-activation of RFs with particular geometrical configurations such as collinearity, leading to linear correlations between non-overlapping collinear RFs (the tilted bowtie shape of Fig. 2D), but not parallel RFs (Fig. 2C). Natural images are also spatially heterogeneous: different image regions can elicit different levels of dependence between RFs outputs [45]. Extreme examples are regions involving single objects with a uniform texture (homogeneous patches) which show a strong variance dependence (Fig. 2E), as opposed to regions spanning multiple objects or objects and background (heterogeneous patches) for which the dependence is weaker (Fig. 2F).

We extended a well-known probabilistic model of the variance dependence of Fig. 2C,D (the Gaussian Scale Mixture, or GSM; [46]) to capture the variability across image regions exemplified in Fig. 2E,F. The GSM describes an RF output, k, as a random variable obtained by multiplying a Gaussian variable (i.e. a random variable that is Gaussian distributed), 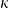, and a second random variable that takes only positive values, 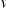, also called the *mixer*.
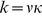
(1)The mixer 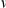 in the model can be shared between multiple RFs (we then describe these RFs as being co-assigned to the same mixer), and can therefore generate statistical coordination, and can be intuitively thought of as representing a relatively global image property, such as contrast, that changes smoothly across space. In contrast, the Gaussian variable 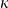 in the model is local to each RF. Consider the case of two RFs, (*k_1_*, *k_2_*), whose respective Gaussian variables are multiplied by a common mixer 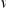. Then the coordination is generated in the following way: in a specific instance where 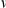 has a large value, both Gaussian variables are multiplied by a large value and therefore *k_1_* and *k_2_* are more likely to be large together. Conversely, when 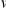 takes a small value, *k_1_* and *k_2_* will likely be small together. This generates precisely the type of dependency observed in Fig. 2C,D. The tilt of the bowtie evident in Fig. 2C comes from linear correlation between the Gaussian variables, captured by their covariance matrix **C**.

We then introduced a variant of the GSM (see also [37]–[39], [47]) that could capture both dependent and independent RFs outputs. Since we were interested in center-surround effects, we designed the model to approximate variance dependencies between a center group of RFs and a surround group, using a combination of: 1) a standard GSM for the case in which the center and surround groups are dependent (they are co-assigned to a common mixer, and can be linearly correlated); and 2) an independent GSM model, whereby the center and surround do not share a common mixer and lack linear correlation. This model, which combines the two extreme cases, is technically a Mixture of GSMs (MGSM [39], [47]).

### Implementation details and model parameters

We implemented the model with a bank of 72 bandpass oriented filters or RFs taken from the first level of a steerable pyramid [44], [48] (see Text S1). We used 8 RFs in a central position (center group, whose outputs are denoted in the following by **k**, and the corresponding Gaussian variables by 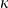) comprising 4 orientations, 0, 45, 90 and 135 degrees from vertical, each at 2 phases. The inclusion of multiple orientations in the center group was motivated by the strong variance dependence typically observed across oriented RFs at a fixed position; it also guaranteed local contrast normalization of the model responses (see below) as commonly assumed in divisive normalization [41]. The 2 phases correspond to a pair of even- and odd-symmetric RFs (quadrature pair). For the surround we used 64 RFs (whose outputs are denoted in the following by **S**, and the corresponding Gaussian variables by 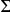) comprising 4 orientations, 2 phases, and 8 positions on a circle surrounding the center RFs with radius 6 pixels. We organized the surround RFs into 4 separate groups, each including all RFs of a given orientation. RFs had peak spatial frequency of 1/6 cycles/pixel, and a diameter of 9 pixels and were partly overlapping. The orientation bandwidth was chosen to approximate the median value found in V1 by Ringach et al. ([49] 23.5 degrees half-width at 70% height of the tuning curve). The qualitative character of the results did not change when using different bandwidths. We included multiple surround groups to guarantee that all orientations were treated equally.

**Figure 3:**
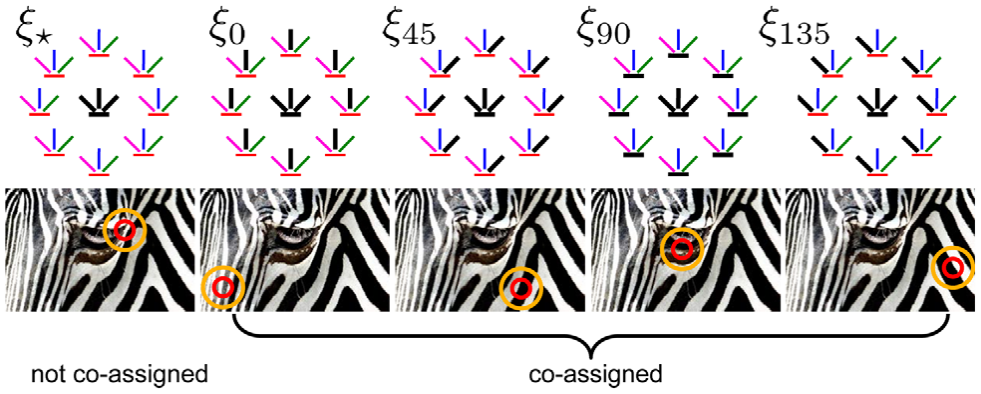
Center-surround configurations corresponding to the different mixture components of the model. We used a bank of linear filters, depicted as colored bars in the top row, comprising 4 orientations at 1 center position and 8 surround positions. We grouped surround RFs according to their orientation, each labeled with a different color. A surround group can be either co-assigned with the center group (i.e., the model assumes dependence between center and surround groups, and includes them both in the normalization pool for the center, as in Fig. 2-top), or not co-assigned (i.e. the model assumes independence between center and surround groups, and does not include the latter in the normalization pool, as in Fig. 2-bottom). The leftmost column depicts the configuration (denoted by 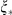) in which none of the surrounds is co-assigned with the center; 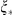 best describes image patches such as those identified by the circles in the bottom row (red and orange circles denote center and surround respectively, as in Fig. 1). The second column depicts the configuration (denoted by 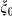) in which the vertical surround is co-assigned with the center; black bars (top) identify the co-assigned groups, circles (bottom) the image patches best described by 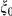. The same conventions are used in the remaining columns.

Fig. 3 illustrates the spatial layout of the RFs, and their structural dependencies in the different mixture components. The leftmost panel depicts the component (denoted by 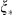) in which none of the surround groups is co-assigned with the center (each RF group in the surround has a different color). The remaining four components (denoted by 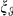 for 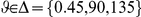) comprise the cases in which all the center RFs, and just those surround RFs that favor orientation 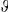, are co-assigned to a common mixer. For example, under 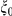 the same mixer multiplies all the center Gaussian variables and the Gaussian variables for the vertical surround, and the corresponding RFs are co-assigned (indicated by black color). Note that even when the center and surround are independent, we still assume that the RFs in the surround share the same mixer, an approximation that was needed for computational tractability (see Discussion).

**Figure 4:**
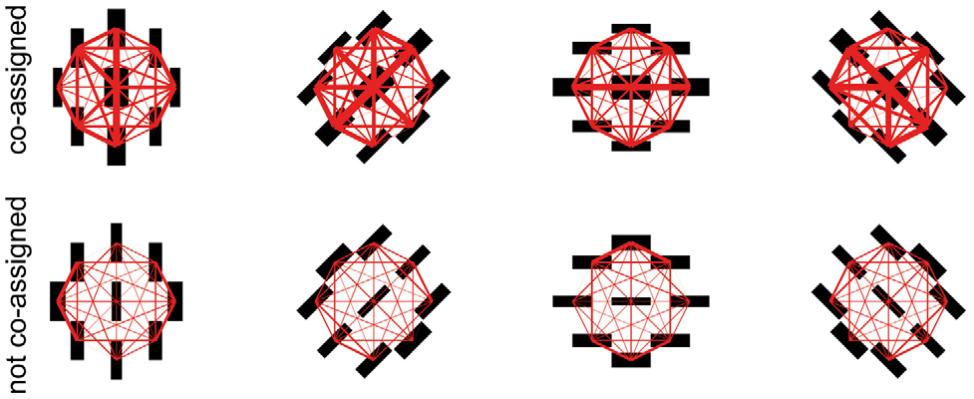
Visualization of the covariance matrices learned from scenes between the Gaussian variables associated with center and surround RF outputs in the mixture components of **Fig. 3**. Top row: co-assigned components (from left to right, 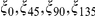); bottom row: independent component (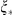). Black bars denote the orientation and relative position of the RFs; bar thickness is proportional to the variance. The thickness of the red lines connecting pairs of bars is proportional to the absolute value of the covariance. For each surround group, we show only the covariance with the center of the same orientation; the covariance with center RFs of different orientations is one to three orders of magnitude weaker. Similarly, we show only the covariances between RFs with even phase; the covariances for the odd phase are similar, while those across different phases are negligible. In each mixture component the variances of the center RF and its collinear neighbors, as well as the covariance between them, are larger reflecting the predominance of collinear structure in scenes.

The parameters governing RF interactions – i.e. the covariance matrices and the prior probability of each component of the MGSM – were optimized by maximizing the likelihood of an ensemble of natural scenes (downloadable from http://neuroscience.aecom.yu.edu/labs/schwartzlab/code/standard.zip). Mathematical details are provided at the end of Materials and Methods. Fig. 4A depicts the resulting covariance matrices. Notice that in the co-assigned components (top row) but not in the independent component (bottom row), the model found larger variances for each central RF and its collinear neighbors, and larger covariance between them, than for its parallel neighbors. For all the results in the paper except where noted, and in Text S4, we renormalized the covariance matrices to make them identical under rotation of the spatial configuration of RFs: e.g., the covariance between the vertical central RF and its collinear neighbors (vertical, above and below the center) under 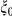, was forced to be the same as the covariance between the horizontal central RF and its collinear neighbors (horizontal, left and right of the center) under 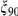. In practice, the effect of the renormalization is to guarantee that model responses corresponding to different RF orientations are identical when the respective input stimuli are rotated copies of each other.

However, to explore the effects of cardinal axis biases and variability across different scenes, we also trained the model separately on each of 40 natural images from the Berkeley Segmentation Dataset (http://www.eecs.berkeley.edu/Research/Projects/CS/vision/bsds), and did not renormalize the covariances. The training was repeated 3 times for each image from random starting points. Training on individual images converged to similar values each time, except for one image on which it did not converge. This image was therefore excluded from further analysis.

### Relating inference in the model to V1 responses

To relate the model to contextual modulation in the visual cortex we assume that firing rates in V1 represent information about the Gaussian variables 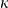 associated with the center RFs. We choose 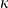 because it represents the local structure of the RFs, which is what contains specific information about the local input image patch. By contrast, 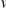 encodes more general information, i.e. the average level of activity in a neighborhood of RFs across orientations and spatial locations.

The MGSM is a statistical model of images, which is sometimes called a generative or graphics model [50]. The task as a whole for vision is to invert this model – using Bayes rule to find the posterior distribution over 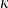 and 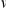. However, here, we compute just the expected value of the Gaussian variables, which we assume to be related to V1 firing rates (via Equation 3, below): in the rest of the paper we also call such estimates the *model responses*.

In practice, we obtained model responses in two steps, i.e. we first collected linear RFs outputs with a given input stimulus, and then we used them to compute the Gaussian estimates; this is an abstract schematization of how V1 firing rates are produced, conceptually similar to the canonical linear-nonlinear scheme [51], and does not imply that the two steps are actually preformed by separate V1 mechanisms. We will address in the following section the relation with divisive normalization and the possible neural mechanisms underlying the computations presented in the remainder of this section.

For a given input stimulus, we first collected the outputs of center RFs **k** and surround RFs **S**. We then computed the expected value 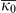 of the Gaussian variable corresponding to a center RF (e.g., tuned to vertical):
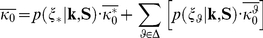
(2)Equation 2 comprises the sum of the estimates under each of the mixture components (respectively denoted 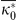 and 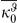), weighted by the posterior probability of the components, 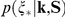 and 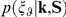. In the first term the surround is not co-assigned. The second term sums over each of the components corresponding to the co-assigned surround orientations. We derived an analytical form for both the mean estimates and the posterior probability, as described below. Eventually, we combined the estimates (Equation 2) for the two phases of the RF (denoted by 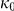 and 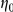), to obtain orientation tuned, phase invariant responses:
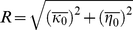
(3)We obtained the mean estimates under each of the components as follows. In the GSM, given knowledge of the value of the mixer 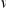, one can obtain 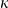 by definition as 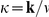. However, the visual system does not know the value of 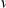, and so can only perform statistical Bayesian inference using information about the prior distribution of 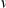. We chose a Rayleigh prior for the mixer variables which gave analytically tractable solutions; however, the qualitative behavior of our simulations would be the same for a range of priors (similar to [37], [47]; and see also priors in [46]). This choice resulted in the following estimates for the co-assigned components:
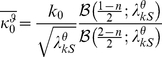
(4)and similarly, we obtained the estimate for the component in which the surround is not co-assigned:
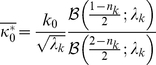
(5)where *n_k_* represents the number of RFs in the center group, and *n* the total number of center and surround RFs. The terms denoted by 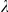 are generically defined as follows:
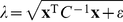
(6)where, in 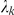, **x** is to be replaced with the center filter outputs and ***C*** is to be replaced with 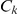; and in 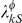, **x** is to be replaced with the center filters and the surround filters with orientation 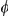, and ***C*** is to be replaced with 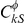. We introduced a small constant ε which sets a minimal gain when the filter activations are zero, to prevent infinities in Equations 4,5 when the RF outputs are zero. In all the simulations, 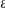 was set to a value (10^−10^) several orders of magnitude smaller than the smallest value observed for the other term under the square root. 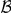 is the modified Bessel function of the second kind, and the ratio of Bessel functions diverges at 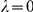 and asymptotes to 1 at infinity, at a rate that depends on *n*; it remains approximately constant over the range of values of 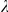 in all our simulations.

Eventually, we inferred the posterior probability, 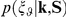, that the center group shares a common mixer with the surround group labeled 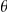, using Bayes rule:
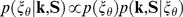
(7)and substituting the first term on the r.h.s. with the learned co-assignment prior, and the second term with its analytic form (see below, Equation 10).

### A generalized form of divisive normalization

The inferred model responses (Equations 2,4,5) thus constitute a form of divisive normalization [40]–[42], where the terms 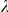 in the denominator represent the normalization signal, and the RFs that contribute to 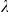 form the normalization pool. It has been shown that divisive normalization has the effect of reducing the higher-order dependencies illustrated in Fig. 2C,D, and accounts for some data on V1 surround modulation [30].

However, our model generalizes standard divisive normalization in two substantial ways. First, consider the effect of covariance. Normalization allows cells to discount a global stimulus property that is shared across RFs, i.e. contrast in simple divisive normalization, or the mixer value in the GSM. The mixer corresponds to RFs that are statistically coordinated and tend to be high or low together in their absolute value. However, in the generative model, large outputs from RFs that often respond together (i.e. RFs with large covariance or linear correlations) could be generated either by a large value of the mixer or by similar draws from the correlated Gaussians; whereas similar, large outputs from RFs that rarely respond together (small covariance) are more likely to have been generated by a large value of the mixer. Therefore linearly correlated RFs should contribute less to the estimate of the mixer (which is loosely proportional to the normalization signal). This arises in the model since the covariance matrices learned from scenes weight the contribution of the RFs to the normalization signal 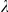 (Equation 6). For instance, a pair of RFs with large variances and large covariance between them (often leading to negative values in the corresponding off-diagonal term of **C^−1^**) exerts less normalization than a pair of RFs with the same variances but small covariance. In addition, an RF with large variance (corresponding to small diagonal terms in the inverse covariance) weights less than one with small variance. In the visual cortex, the effect of such weights may be represented indirectly in the strength of excitatory connections that are set with development; indeed the higher correlations, in the model, between overlapping RFs, as well as non-overlapping collinear RFs, are qualitatively in agreement with the known specificity and anisotropy of horizontal and feedback connections [52]–[58] (see also Discussion).

Second, Equation 2 uses a stimulus-dependent normalization pool, since, for any given input stimulus, only RFs that are inferred as being statistically coordinated and thus to share a common mixer, are jointly normalized (the normalization pool comprises different RFs for each mixture component in Equations 4,5). The same RFs can be coordinated for some stimuli and not for others.

The computation involved, which in the model is distinct from the evaluation of the corresponding normalization signals (Equation 6), does not necessarily have to be segregated in the biological system, and may be achieved by a number of neural mechanisms. One possibility may be that the normalization signals are computed by inhibitory interneurons that pool the outputs of distinct subpopulations: the different firing thresholds or diversity across types of interneurons [59], [60] have been previously indicated as mechanisms that may control whether or not surround inhibition is active on a given input. A complementary view is that surround modulation is an emergent property of the cortical network; in this scenario, the strength of the surround influence may be determined by stimulus-dependent switching between cortical network states [61] or changes in functional connectivity [62], or by the exact balance between excitatory and inhibitory conductances, which are known to change in parallel with surround stimulation [63].

**Figure 5:**
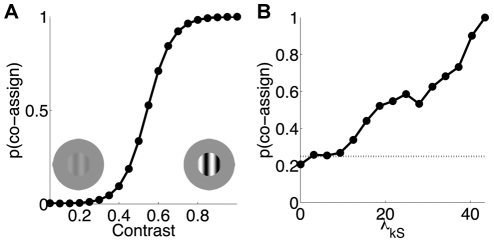
Co-assignment probability depends on image contrast. (**A**) Probability for the vertical center and surround RFs as a function of grating contrast. The stimulus diameter is 7 pixels. All the RFs have diameter 9 pixels, and the mid point of each surround RF is located 6 pixels away from the mid point of the center RF, so a stimulus 7 pixels wide encroaches on the surround RFs. Although it does so only by 2 pixels, in the simulations this leads to a large co-assignment probability at high contrast; we have verified that this is not the case for a stimulus 5 pixel wide (in which case the overlap with surround RFs is even smaller, and not enough to recruit the surround). (**B**) Probability for the vertical center and surround RFs on natural images. The X axis represents values of 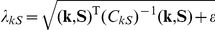 for vertical RFs (**k**, **S**), with covariance matrix 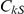. The term 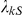 therefore increases for larger values of the RF outputs (note that for any fixed image, scaling the contrast by a factor *c* also scales 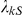 by *c*). For each input image, we computed 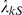 and the co-assignment probability for the configuration 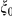; the Y axis represents the mean of such probability across the inputs corresponding to given values of 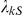. The dashed line corresponds to the prior probability learned by the model on natural images.

For a given input stimulus, we inferred the posterior co-assignment probabilities of each mixture component, 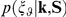, using Bayes rule (see Equation 7). These probabilities measure how well each component explains the input data. Intuitively, the probability is large when the stimuli in the center and surround are similar (e.g. for gratings of similar orientation and contrast), and for a given stimulus, it tends to be larger at high contrast as illustrated in Fig. 5. More precisely, 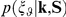 is a function of the center and surround RFs outputs, combined using the corresponding covariance matrices as in Equation 6. In our implementation, for a given input stimulus the probability of co-assignment does not vary across center orientations (because we grouped together all center RFs with different orientations). The surround RFs are grouped together according to their orientation, and so all RFs within one surround group have the same probability of being co-assigned with the center, but each surround group has a different probability (e.g. for a large vertical grating, the vertical surround has high probability, while the horizontal surround has low probability). On each given stimulus, the probabilities across the 4 surround groups, plus the probability of no assignment (i.e. that no one of the surrounds is co-assigned with the center), jointly add up to 1.

**Figure 6:**
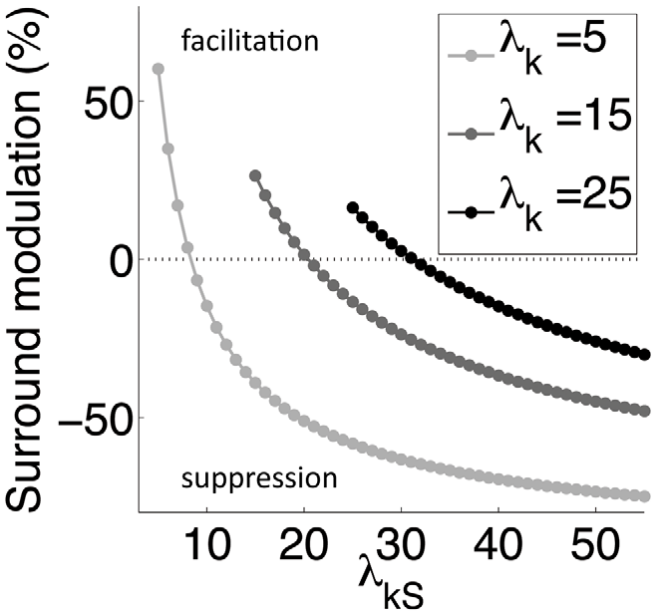
The model encompasses both surround suppression and facilitation. Modulation of simulated model responses when the surround is co-assigned (*R_co-assign_*; e.g. with a large, homogeneous grating) relative to when it is not co-assigned (*R_no-assign_*; e.g. with a grating smaller than the central RF). Percent surround modulation is computed as 100*(*R_co-assign_−R_no-assign_*)/*R_no-assign_*. This quantity depends on RFs outputs only via 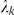 and 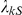. Since 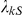 appears in the denominator of Equation 3, the larger the 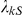 the smaller the model response. We choose three representative values of 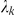 (note that 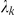 increases with larger center RF output). Surround modulation in the model can facilitate center responses for weak surrounds (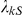 comparable with 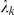), and gradually switch to suppression as surround strength (and therefore 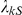) increases (relative to 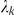). No surround modulation (dashed line) is observed e.g. with small stimuli or large, non-homogeneous stimuli for which center and surround RFs are not co-assigned.

Note also that, differently from standard divisive normalization which is inherently suppressive, our model encompasses both surround suppression and facilitation as summarized in Fig. 6: a strongly driven surround suppresses center responses, whereas a weakly driven surround facilitates, and the relative modulation is larger when the center RF is weakly driven.

### Model equations

The full distribution of the RFs variables under the MGSM is given by the mixture model:
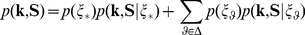
(8)in which the terms 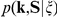 represent the joint distribution of the center and surround RF outputs **k** and **S** under each of the five possible mixture components, weighted by their prior probabilities 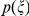. The terms 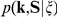 can be derived analytically as follows. First, we assumed a Rayleigh prior on the mixer variables:
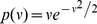
(9)Under the configuration 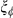 in which the surround of orientation 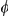 is co-assigned with the center, we can exploit the independence among groups that do not share a mixer, and obtain:
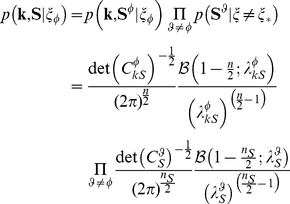
(10)where, in the first line, 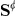
denotes the surround RFs with orientation 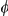, and similarly for 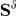. In the second line the factors are derived as in a standard GSM, as detailed in [37], [47]; *n_k_* and *n_S_* represent the number of RFs in the center and surround groups respectively, and *n* = *n_k_*+*n_S_*; 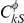 is the covariance matrix among the center group and the surround group with orientation 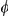; 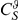 is the covariance of the surround with orientation 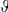; 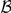 is the modified Bessel function of the second kind. The terms denoted by 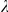 are defined as in Equation 6.

Similarly, we can derive the distribution conditional on 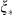, namely in the case that all groups are mutually independent:
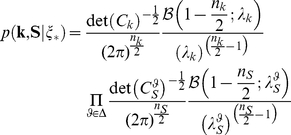
(11)where 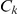 is the covariance matrix of the center Gaussian variables, and 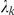 is defined as in Equation 6.

### Model training

The parameters governing RF interactions – i.e. the covariance matrices and the prior probability of each component of the MGSM – need to be optimized for an ensemble of natural scenes. The training data were obtained by randomly sampling 25000 patches from an ensemble of 5 natural images from a database of standard images used in image compression benchmarks (known as Einstein, boats, goldhill, mountain, crowd; see Fig. S1; the images are downloadable from http://neuroscience.aecom.yu.edu/labs/schwartzlab/code/standard.zip). The parameters to be estimated were the covariance matrices associated to the different components of the model, collectively denoted by 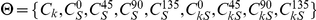, as well as the prior probability of each component, denoted by 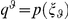 and 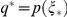. The model learned the full covariances, including the terms coupling opposite RFs phases which were typically close to zero. To find the optimal parameters we maximized the likelihood of the data under the model; we implemented a Generalized Expectation-Maximization (GEM) algorithm, where a full EM cycle is divided into several sub-cycles, each one involving a full E-step and a partial M-step performed only on one covariance matrix. In the E-step we computed an estimate, 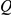, of the posterior distribution over the assignment variable, given the RF responses and the previous estimates of the parameters (denoted by the superscript *old*). This was obtained via Bayes rule:
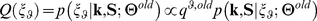
(12)and similarly for 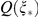. Then in the M-step we adopted conjugate gradient descent, to maximize the complete–data Log Likelihood, namely:
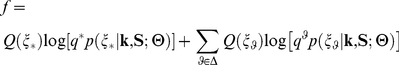
(13)The training was unsupervised, i.e. we did not pre-specify the co-assignments of the training set, but rather let the model infer them at each E-step. The EM algorithm is not guaranteed to find a global maximum; however, repeated training runs with different randomly chosen starting points produced convergence to similar parameter values. More details are provided in Text S2 (see also [47]).

## Results

Our hypothesis, illustrated schematically in Fig. 1, is that contextual modulation in V1 is sensitive to the statistical homogeneity of the visual inputs; or, more specifically, that it is engaged by homogeneous stimuli (e.g. an image patch entirely embedded within a single object, such as the one on the right in the figure), but not by heterogeneous stimuli (e.g., an image patch that includes different objects, such as the one on the left). In order to test the hypothesis, we introduced a model of spatial dependencies in natural images that formalized this intuitive notion of homogeneity. The model used a mixture of two components, which describe respectively image patches that elicit statistically independent, or dependent, RFs outputs across center and surround locations. We then related correct (i.e. Bayesian) inference in the model (specifically, the expected value of the local Gaussian variable; Equations 2,3), to the firing rates of V1 neurons. This resulted in a divisive form of surround modulation that is related to descriptive [40], [41] and statistical [30] models, but substantially extends them. Below, we provide further intuition into two key features of the model; then, in the following sections, we compare the model simulations to experimental data on V1 firing rates and perceptual effects.

First, the model uses a normalization pool (i.e., the group of RFs whose outputs contribute to the normalization signal) that depends on the input stimulus (see Equations 2,4,5): when center and surround RFs outputs are statistically coordinated (i.e. they lead to high posterior co-assignment probability, Equation 10), then both are included in the normalization pool for the center; whereas, when they are independent, the surround RFs are excluded from the normalization pool. For a given input stimulus, we measured the degree of homogeneity (and therefore involvement of the surround RFs) by the probability of co-assignment. Intuitively, this probability is larger for stimuli that elicit similar outputs for center and surround RFs; and this indeed turns out to be true of Bayesian inference in our model. In addition, the probability tends to be larger for high contrast. The grating patch of Fig. 5A provides a simple, specific example, showing that the probability for the vertical surround RFs increases monotonically with image contrast. We also explored the co-assignment probability as a function of the combined outputs of both center and surround RFs (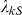; see Equation 6) with natural image patches. Fig. 5B shows that on average the probability increases with 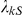 (note that for any fixed image, scaling the contrast by a factor *c* also scales 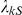 by *c*).

Second, the linear correlations between center and surround RFs, captured by their covariance matrix **C** when the surround is co-assigned, affect the normalization signal, since in 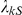 the RFs outputs are weighted by **C** ^−1^ (see Equation 6). Thus, the normalization signal can be reduced, and the model response enhanced, when the covariance between RFs is large or when a given RF variance is larger (as described in Materials and Methods). This, as shown below, plays an important role in the perceptual effects related to collinear facilitation. In addition our model encompasses both suppressive and facilitating surround influences – in this it is different from standard divisive normalization models, which are inherently suppressive. In Fig. 6 we compared model responses to simulated inputs (i.e., preset values of 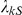 and 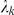), when the surround is not co-assigned (e.g. as would be obtained with a grating smaller than the central RF) relative to when it is co-assigned (e.g. when the same grating is expanded beyond the central RF). We observed suppression for stimuli that drive the surround strongly (i.e., large values of 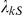 for fixed 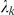); decreasing the surround strength led to a gradual reduction of the suppression, which eventually turned into facilitation for stimuli that drive the surround weakly (i.e., small values of 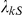 for fixed 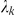). By definition, no surround modulation (dashed line) is observed e.g. with stimuli much smaller than the RF, or large non-homogeneous stimuli, for which center and surround RFs are not co-assigned.

### Contrast dependence of RF size

We learned the parameters of the model (i.e., the covariance matrices and the prior co-assignment probabilities) entirely from the natural scenes, and then fixed them. We first evaluated the model by comparing its response (Equation 3) to presentations of different forms and sizes of sinusoidal gratings with those described in previous neurophysiological studies of spatial contextual modulation. Physiology experiments have made extensive use of gratings to show that stimuli presented in regions of visual space that do not drive the neuron (i.e. the surround) can still strongly modulate the responses to a stimulus presented within the RF.

**Figure 7:**
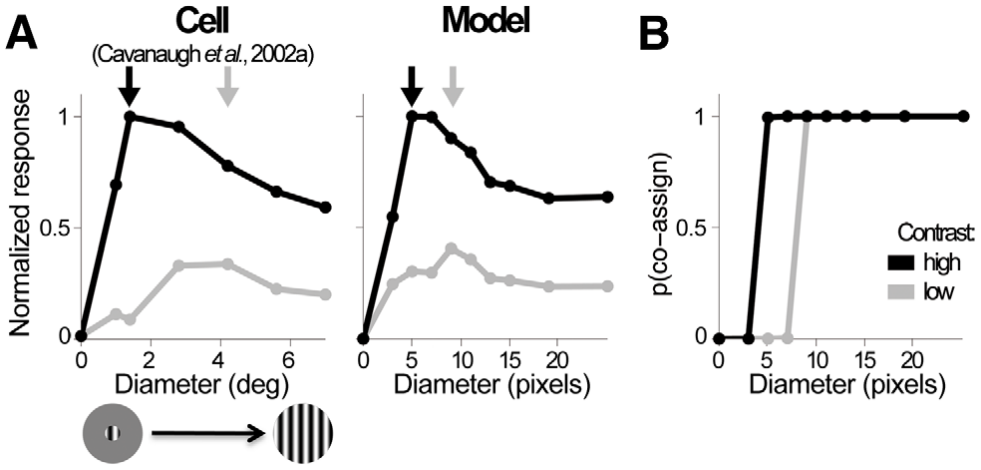
Expansion of receptive field size at low contrast. (**A**) Normalized mean response rate of a V1 neuron (left; [8]) and the model (right) as a function of stimulus size at two contrasts. The largest size we used to test the model covers the full extent of the surround RFs, and larger sizes will not change RFs outputs; for the 5 largest sizes tested, we observed little change in surround RFs outputs and model responses. Stimulus orientation and spatial frequency are optimal for the neuron. The insets show some example stimuli. The arrows (gray, low contrast; black, high contrast) indicate peak response diameter - or RF size. (**B**) Probability that center and vertical surround RFs are co-assigned to the same normalization pool, and therefore contribute to the divisive normalization of the model response; the stimuli are the same as in (**A**). At the smallest non-zero size, surround RFs are silent and therefore the surround is not co-assigned. At intermediate sizes, surround RFs outputs are weaker than center RFs outputs and co-assignment probability increases with contrast, as illustrated in Fig. 5. At the largest sizes, RFs outputs are similar between center and surround and the surround is co-assigned.

First, we tested gratings of variable size and contrast whose orientation and spatial frequency match the chosen RF. Experiments in cat and macaque monkey V1 [8], [64], [65] show that at fixed contrast, neural responses typically increase as a function of stimulus size up to a peak value, and then decrease for larger stimuli that recruit the suppressive surround. The peak responses correspond to larger diameters at low than high contrasts (Fig. 7A-left; the studies cited above reported an average expansion factor across the population in the range 2.3 to 4). Fig. 7A-right shows the similar behavior of our model, including a contrast-related expansion of similar magnitude from high to low contrast in our model. For intermediate contrasts we observed a gradual peak shift.

The contrast-dependence of size tuning has previously been ascribed to divisive normalization [30], [35]. Cavanaugh et al. [8] showed that a divisive model with variable gains for the center (numerator) and surround (denominator) accounted well for the contrast-related expansion; model parameters obtained from data fitting showed that the relative influence of the surround increased with contrast, suggesting that the inhibitory surround is less sensitive than the center at lower contrasts (but see also [62], that supported the alternative hypothesis that low contrast enhances recurrent excitatory interactions). In our model, the flexible assignment process provided a related, but statistically motivated explanation of the expansion. For gratings of intermediate sizes that covered the surround only partially, the co-assignment probability was larger when the contrast was high than when it was low (Fig. 7B; see also Fig. 5). Therefore larger stimuli were necessary to recruit surround modulation at low than high contrast. This pattern of assignments was obtained directly from statistical inference rather than from data-fitting constraints, and did not depend on any asymmetry between center and surround.

### Orientation tuning of surround modulation

**Figure 8 pcbi-1002405-g008:**
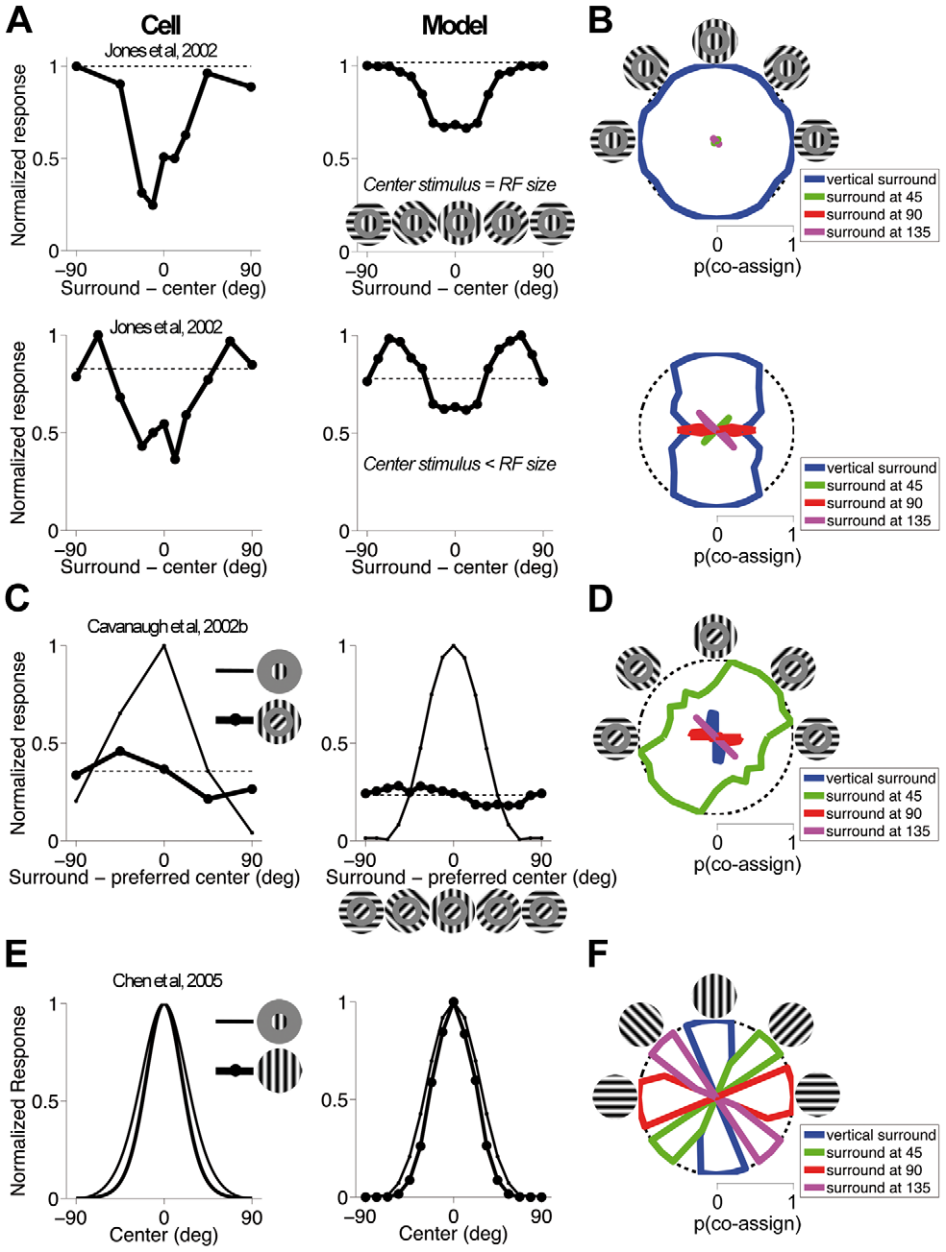
Orientation tuning of surround modulation. (**A**) Normalized mean response rate of V1 neurons (top and bottom left; [10], their Fig. 1A and 4D, respectively) and the model (right) to mid contrast stimuli comprising an optimally oriented grating presented to the RF center, and an annular grating of variable orientation in the surround. The insets show some example stimuli. The outer diameter of the annular patches used to test the model covers the full extent of the surround, and the inner diameter (11 pixels) is larger than the center RF to ensure that the surround stimulus does not encroach on the center. In the top-right panel, the center stimulus equals the center RF diameter (9 pixels), in bottom-right panel it is smaller (5 pixels). Responses (circles) are plotted as a function of the difference between center and surround orientation. The dashed line denotes the response to an optimal grating patch not surrounded by an annulus. (**B**) Probability that center RFs and surround RFs of each orientation (colors indicated in the legend) are co-assigned to the same normalization pool; the stimuli are the same as in (**A**), and some examples are depicted in the icons. The bounding circle (dashed line) represents a probability of 1. Probabilities are plotted in polar coordinates: angular position represents the orientation of the surround stimulus; distance from the origin represents the probability. (**C**) Circles and thick lines: Normalized mean response rate of a V1 neuron (left; [9]) and the model (right) to stimuli similar to (**A**), except that the central grating is tilted 45 degrees away from the neuron's preference. Thin lines: Orientation tuning curves measured with a small grating. (**D**) Probability that center RFs and surround RFs of each orientation are co-assigned. The conventions are the same as in (**B**). (**E**) Orientation tuning curves of a V1 neuron (left; [11]) and the model (right) measured with small gratings confined to the center RF (thin line), and large gratings covering also the surround (thick line and circles): narrower tuning curves are observed with large stimuli. Each curve is normalized by the response to the optimal orientation. In the experiment, the diameter of large gratings is 5 times the center RF size; in our simulations, we used large gratings that cover the full surround extent. (**F**) Probability that center RFs and surround RFs of each orientation are co-assigned, for the large gratings. For each stimulus orientation, the surround group with orientation closest to the stimulus is co-assigned with center RFs.

We next addressed three sets of data related to the orientation tuning of surround modulation (Fig. 8), for which the involvement of multiple surround normalization pools in the model provided a novel, potentially unifying explanation.

We first verified that the model produces contrast-invariant orientation tuning curves (Fig. S2). We then assessed how a surround annular grating modulated the response to a fixed, optimally-oriented central grating, as a function of their orientation difference. Without loss of generality, we refer to the central grating as being vertical, both for the data and the model. Several studies, e.g. [7], [9], [10], [66]–[68], reported that the strongest suppression (relative to the response to the center grating alone) occurred when the center and surround were similar in orientation, while large orientation differences led to little or no suppression, as exemplified by the cell in Fig. 8A-top-left [10]. Our model reproduced these features of surround orientation tuning (Fig. 8A-top-right).

We also explored the model behavior as a function of the stimulus contrast and the sizes of the central grating patch and surround annulus (see Text S3). Generally speaking, in the model we observed surround facilitation when the center was not optimally stimulated, and the surround was weakly driven and co-assigned (Fig. 6). For instance, when we introduced a gap to make the center patch smaller than the center RFs, and with the surround annulus covering only part of the surround RFs, we found facilitation for large orientation differences between center and surround. The response was maximized with annuli tilted less than 90 degrees relative to the center (Fig. 8A-bottom-right). These conditions have not been explored systematically in experiments, but some evidence for the above effects was reported by studies that tested surround orientation finely (e.g., Fig. 8A-bottom-left, reported in [10] using mid contrast; [67] using low contrast in the center and high in the surround), and recent models of the perceptual tilt illusion have implicated facilitation from non-orthogonal surrounds [69], [70]. On the other hand, other experimental studies have not reported facilitation at all [7], [68], and [8] reported that an iso-oriented surround stimulus is always suppressive, regardless of the contrast of the center and surround stimulus. Our model partly failed to reproduce this observation, since it produced facilitation when we combined small center patches with large gap sizes at low contrasts (see Discussion; see also Text S3). The dependence of surround tuning on contrast, and more generally on how strongly the RF is driven, has yet to be explored more systematically.

The responses of the model (Fig. 8A-right) depended on the interaction between different surround components as follows (Fig. 8B; see also Text S3 for more examples). In the top row, the center grating patch partly activated surround RFs, leading to a high co-assignment probability for the vertical surround, regardless of the annulus orientation: the strength of the suppression then simply depended on how much the surround annulus increased the outputs of the vertical surround RFs. In the bottom row, we reduced the size of the center stimulus. In this case, for stimuli with small orientation differences (less than 45 degrees) between the (vertical) center and the surround we observed large co-assignment probability for the vertical surround group: in this regime, surround modulation was suppressive. Modulation switched to facilitation as the orientation difference increased and surround strength decreased (corresponding to reducing 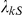 for fixed 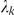 in Fig. 6). At much larger orientation differences, the co-assignment probability decreased for the vertical surround group, but increased for the surround groups oriented similarly to the annulus, leading to less, and eventually no, facilitation. The model response thus combined, via Equation 2, facilitation from the (weakly driven) vertical surround with the suppressive influence of the (strongly driven) diagonal and horizontal surrounds.

Interestingly, the presence in the model of multiple surround groups also allowed it to reproduce data on surround modulation of a non-optimal center stimulus (Fig. 8C). With this type of stimulus, several groups reported that maximal suppression occurs more often when the surround is oriented similarly to the center, than when the surround is oriented similarly to the cell's preference [9], [10], [54], [71]. For the simulations we fixed the orientation of the central patch to 45 degrees from vertical, but continued to report the posterior mean of *k_0_*. Model responses showed the same effect, since stimuli with small orientation differences between center and surround led to large co-assignment probability for the surround group oriented 45 degrees from vertical (Fig. 8D), even though this orientation differed from the vertical orientation on which the model's response was based. To our knowledge, the result of Fig. 8C has not previously been modeled. Our statistical explanation is based on the possibility of using several, differently tuned normalization pools.

Finally, we compared the orientation tuning curves measured with small gratings confined to the center RF, and large homogeneous (single orientation) gratings that also covered the surround. Chen et al. [11] reported that increasing the grating's diameter, up to 5 times the center RF size, often leads to narrower tuning curves in V1 (Fig. 8E; Vinje and Gallant [72] previously reported that, with natural images, stimulation of the RF surround enhanced the cells' selectivity). In our simulations, the surround was not co-assigned when the grating was small. For large gratings that covered the full extent of the surround, center responses were normalized by the surround group with orientation closest to that of the stimulus (Fig. 8F). Therefore, when the orientation changed gradually from optimal to orthogonal, the vertical central RF output decreased, but the outputs of the co-assigned surround group remained approximately constant. This led to stronger suppression (relative to the small stimuli) at non-optimal orientations, i.e. a narrower tuning curve.

### Spatial asymmetry of surround modulation

**Figure 9:**
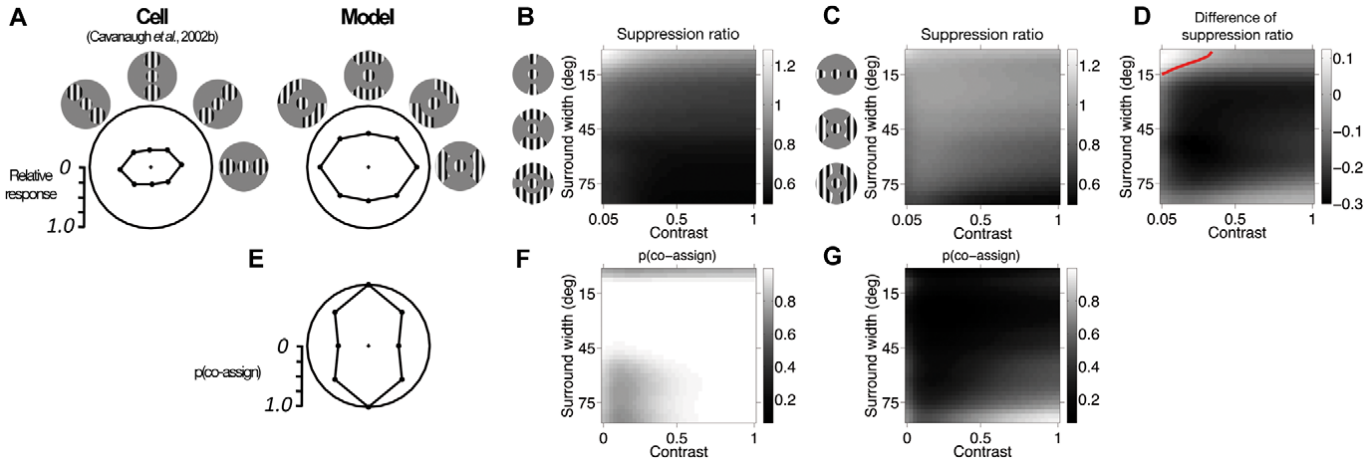
Spatial asymmetry of surround modulation. (**A**) Normalized mean response rate of an example V1 neuron (left; [9]) and the model (right), to optimally oriented stimuli comprising a grating presented to the center of the RF, and peripheral patches confined to specific regions of the surround. Icons depict the stimulus configurations. The bounding circle represents the normalized response to the center stimulus alone. Responses are plotted in polar coordinates: angular position represents the location of the surround stimulus; distance from the origin represents the magnitude of response. In the experiments the size of the surround stimuli is optimized individually for each cell. For the model, we used annular sectors, rather than circular patches: first, this allowed us to explore systematically the changes in responses as a function of the surround stimulus angular size (thus making predictions beyond the original experiment); and, second, this was needed to recruit a sufficient number of surround RFs, given the coarse spatial sampling of the surround (Materials and Methods). (**B,C**) Suppression ratio (coded by pixel intensity) as a function of stimulus contrast and angular size of the peripheral patches (**B** collinear; **C** parallel). Suppression ratio is the ratio between the full-stimulus response and the response to the central grating alone; values greater than 1 denote facilitation, smaller than 1, suppression. (**D**) Difference of suppression ratios (coded by pixel intensity) between collinear and parallel configurations. Negative values imply that the collinear configuration is more suppressive than the orthogonal, and vice versa for positive values; the red contour denotes zero-crossing. (**E**) Probability that center and vertical surround RFs are co-assigned to the same normalization pool, and therefore contribute to the divisive normalization of the model response, for the stimuli of (**A**). (**F,G**) Co-assignment probability (coded by pixel intensity) as a function of stimulus contrast and angular size of the peripheral patches (**F** collinear; **G** parallel).

We then addressed the spatial organization of surround modulation, to illustrate the interplay between the assignment process and the spatial structure of correlations learned by the model from natural images. Different regions of the RF surround can produce different levels of modulation when stimulated with grating patches oriented similarly to the center (called positional bias). Walker et al. [7] and Cavanaugh et al. [9] reported that the location of the surround that elicits maximal suppression varies across the population, but is found approximately 2 to 3 times more often near the end of the RF (a collinear arrangement) than near the sides (a parallel arrangement). The cell of Fig. 9A-left illustrates the positional bias with stronger suppression for collinear stimuli. The model reproduced the same qualitative behavior with similar stimuli (Fig. 9A-right), reflecting a higher co-assignment probability for the collinear arrangement (Fig. 9E) as explained below.

To elucidate the origins of this response of the model, we systematically tested a larger range of contrast and size values than has so far been experimentally examined (Fig. 9B,C). First, consistent with the above, we observed strong suppression for large collinear stimuli at moderate to high contrasts. We also found that collinearity could lead to facilitation under some conditions such as low contrast, thin, stimuli. Conversely, the parallel arrangement produced little modulation, mostly suppressive. Direct comparison of the two arrangements (Fig. 9D) showed that, depending on where the stimuli fell in the contrast/size space, the collinear surround could elicit both larger (low contrast, thin stimuli) and smaller (mid contrast, fat stimuli) model responses than the parallel arrangement. Model simulations across such stimulus space thus offered a specific, testable prediction.

Our model explains the observed spatial asymmetry of modulation by the form of the covariance matrices learned from natural scenes. In the mixture component with dependent center and vertical surround groups, the variances of the vertical center RF and its collinear neighbors were similar, and higher than the variances of the parallel neighbors (Fig. 4A). Recall that the co-assignment probability is a function of the terms 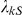, 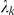 and it is larger when they have a similar magnitude; these terms are computed using the covariance matrices (see Materials and Methods) and were more similar between center and surround for stimuli that covered the center and collinear surround, than for stimuli covering the parallel surround. This made collinear stimuli more likely to be co-assigned (Fig. 9F,G) and modulate center responses. At mid contrast, fat surround, this led to stronger collinear suppression. The sign of the modulation switched to facilitation when we used low contrast, thin collinear stimuli (thinner stimuli produce weaker surround RFs outputs thus reducing 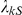 for fixed 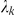; see Fig. 6).

Collinear facilitation with grating stimuli has been observed in V1, although with the contrast not matched between center and surround; for instance, Polat et al. [73] reported that a high contrast collinear surround could facilitate the responses to a low contrast central grating patch, but suppress a high contrast center. Kasamatsu et al. [74] quantified the average modulation across their population for collinear flankers presented at 0.5 and 0.8 contrast, and reported a peak facilitation of approximately 17% when the center contrast was similar to the contrast threshold of the cells, and weak suppression in the range 2–5% for larger center contrasts (up to >10 times the cells' threshold). We observed similar changes in model responses: the average modulation, for flankers presented at contrasts ranging between 0.5 and 0.8, showed 16% peak facilitation at low center contrast (0.2), and suppression in the range 1–6% for larger center contrast (0.35 to 0.8). Interestingly, the model also reproduced “far” surround facilitation (see e.g. large gap sizes in the first figure of Text S3), which was observed in [12] often with low contrast center but only rarely with high contrast center. Our model provides a common explanation for the two phenomena: A low contrast central grating by itself did not recruit surround modulation (see Fig. 5B), but the composite stimuli did. In both experiments, the surround was weakly driven, thus leading to facilitation (corresponding to small 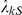 for fixed 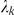 in Fig. 6). Conversely, a high contrast center grating by itself recruited the surround, and so the addition of the surround stimuli only increased the normalization signal, albeit weakly, and therefore became suppressive.

In addition to the effect we discussed, Cavanaugh et al. [7] reported a complementary, although weaker, positional bias when using surround patches orthogonally oriented relative to the center grating: They observed more often stronger suppression from orthogonal patches placed at the side, rather than at the end, of the RF. Our model qualitatively reproduced this finding, as illustrated in Text S4.

**Figure 10:**
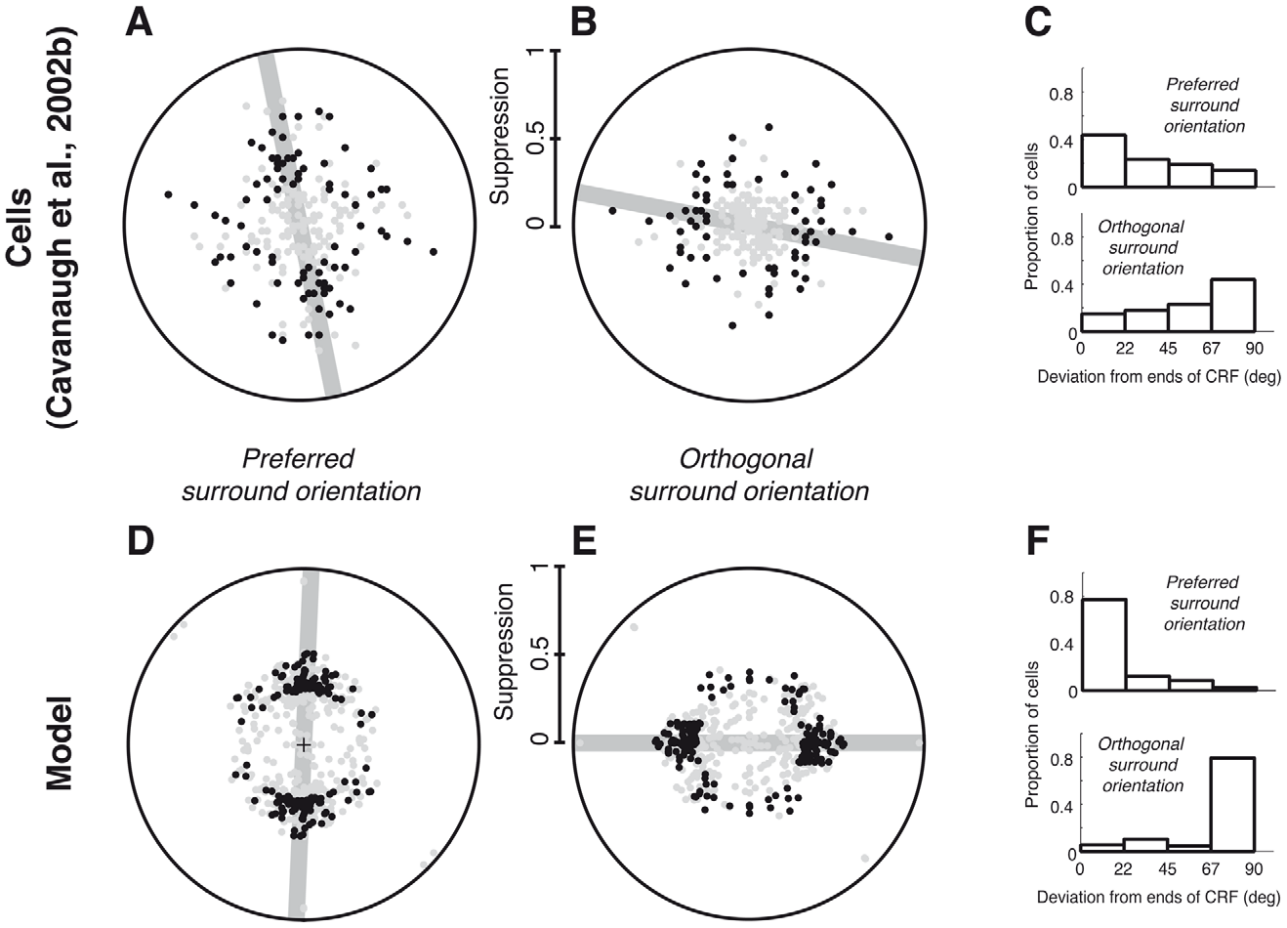
Variability of positional biases. (**A–C**) V1 population data from [9], N = 113. (**D–F**) model simulations for 4 center orientations, 2 contrasts (0.25, 0.5) and 39 parameter sets resulting from different natural image training sets (see Materials and Methods). (**A,D**) were obtained with the surround stimuli of Fig. 9. The magnitude of the suppressive effect and surround location of greatest suppression are plotted in polar coordinates (duplicated around the circle for visualization): points farther from the origin correspond to stronger suppression than those closer to the origin. The location of maximal suppression is the angle of the vector computed from the response reduction, as in [9]. Black circles represent cases with a strong effect (i.e. the magnitude of the orientation bias estimate was at least 0.2 and the maximum suppression was at least 0.3; 42/113 cells in [9] and 83/272 instances of the model matched both criteria). The gray line shows the angle estimate for the cluster of such points. (**B,E**) were obtained with surround stimuli presented in the same locations as in Fig. 9, but oriented orthogonally to the center (see also Text S4). All conventions are the same as in (**A,D**). The effect was strong in 33/113 cells in [9], and 106/272 model instances. (**C,F**) represent, only for the cases with a strong effect, the distribution of the difference between the most suppressive surround location and the axis of preferred orientation.

However, that there is a considerable scatter of positional biases due to cell-to-cell variability in [7], [9] (Fig. 10A–C). We explored in the model whether some of the variability could relate to the properties of natural scenes; note that for these simulations (Fig. 10 and Text S4) we did not impose the rotational invariance of the covariances, as explained in Materials and Methods. First, we found that, due to the predominance of cardinal orientations in scenes, the bias was stronger for the model units with cardinal orientations than with diagonal orientations (Text S4). We then expanded on the previous observation, by optimizing the model on several different natural images separately, and indeed found a large scatter of positional biases (Fig. 10D–F) qualitatively consistent with the data, although to a smaller degree. Our model may be missing an additional source of variability related to scene statistics, due to the constraint of always grouping together iso-oriented RFs at all surround locations: it is possible that a more flexible assignment scheme with multiple mixer pools and RFs across space (e.g. [37]) could capture statistical regularities in scenes beyond collinearity (see also Discussion).

The data presented so far provided an illustration of the biological significance of the two key components of the model, i.e. the assignment and the covariances. As a control, we presented the same stimuli to a reduced model that ignored the assignments and assumed that center RFs were always normalized by the vertical surround group. Such reduced model produced a much weaker contrast-dependence of size tuning of the surround compared to Fig. 7; and it did not produce maximal responses for intermediate orientation differences as in Fig. 8A, nor could it capture the effects of Fig. 8C,E. We also considered a reduced model that assumed diagonal covariance matrices, rather than learning them from natural images. As opposed to Fig. 9, this model produced no difference in co-assignments, nor in surround strength, from different surround locations.

### Perceptual salience

We then asked whether the same statistically motivated computational principles underlie perceptual salience: local image regions are deemed salient when e.g. they can be detected more readily, appear to have higher luminance, or attract human gaze more often compared to the remaining parts of the image (see [15], [20] and references therein). Several models [15]–[18] have related bottom-up salience to the local contrast in image features (e.g., luminance, orientation, color channels). Most pertinently for us, the idea that V1 is a neural substrate of salience processing has been formalized theoretically and supported experimentally [20], [21], [75]–[77].

To address saliency effects, we combined model responses corresponding to all four center orientations, as is typical in saliency computations. Specifically, for a given image, at each input location we computed the responses as in Equation 3 separately for each orientations, and then took the maximum [22]. The result was interpreted as a (statistically-based) salience map.

**Figure 11:**
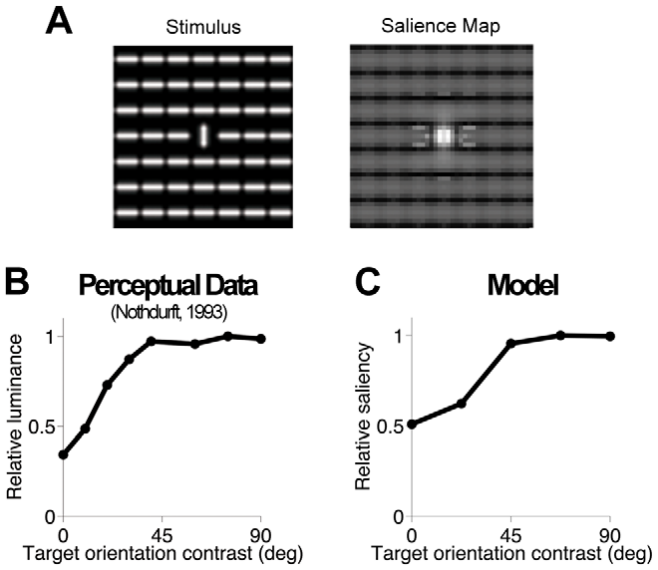
Perceptual pop-out in a population of model neurons. (**A**) Example stimulus (left) and the corresponding salience map (right). Bars are 5 pixels long (i.e., smaller than RFs) and separated by 6 pixels (equal to RFs spacing; see Materials and Methods). Pixel intensity codes for salience, with brighter pixels denoting higher salience. (**B**) Mean perceived luminance [3] of a target defined by orientation-contrast, such as the vertical bar in the icon on (**A**, left). Perceived luminance is defined as the luminance of a luminance-contrast target (i.e. a bar with the same orientation as the background distractors, but higher luminance), required for it to be perceived as salient as the orientation-contrast target. Values are rescaled to a maximum of 1; the relative perceived luminance of the background bars is 0.36. (**C**) Saliency computed by the model for the orientation-contrast targets. Values are rescaled to a maximum of 1; the relative saliency of the background bars is 0.51.

The stimulus shown in Fig. 11A-left illustrates the phenomenon of perceptual pop-out of a central target that differs from a uniform background of distractors by a single feature, in this case orientation. Consistent with the percept, the target has by far the greatest salience in the output salience map (Fig. 11A-right). Nothdurft [3] reported that — with a luminance matching experiment above detection threshold — the relative target saliency saturated with a center-surround orientation difference around 45° (Fig. 11B). By comparison, many salience models exhibit a linear increase in response (see [16] for discussion). Our model correctly captured the form of the nonlinearity, with the specific nature of the saturation depending on stimulus design: with high contrast, 5 pixels long bars, the vertical surround group always normalized the responses at the location of the center bar, and therefore surround bars oriented 45 degrees or more from vertical modulated center responses only weakly (Fig. 11C; see Fig. S3 for examples with different bar contrasts/sizes/separations).

**Figure 12:**
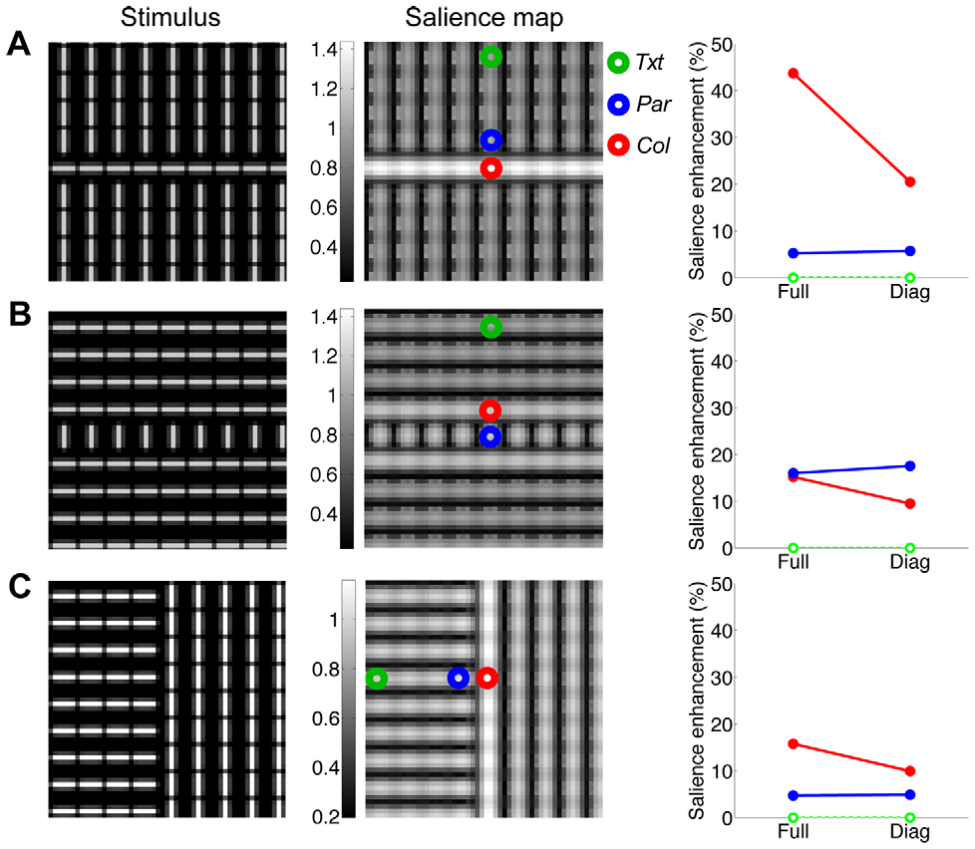
Perceptual saliency of collinear stimuli. (**A,B**) Illustration of the collinear enhancement of salience. Left: Original stimulus. Bars are 5 pixels long and separated by 6 pixels (equal to RFs spacing; see Materials and Methods). Center: salience map (pixel intensity codes for salience, with brighter pixels denoting higher salience). Symbols on the map identify regions of homogeneous texture (*Txt*, green), and the boundary regions formed by collinear (*Col*, red) or parallel (*Par*, blue) bars. Right: salience enhancement (Y axis) is quantified as *(Col-Txt)/Txt* (red line and symbols, for the collinear side) and *(Par-Txt)/Txt* (blue line and symbols, for the parallel side); the enhancement is 0 by definition for the homogeneous texture regions (green line and symbols). On the X axis, *Full* stands for the results obtained with the full model, whereas *Diag* stands for a reduced model that sets the covariance between center and surround RFs proportional to the identity matrix, rather than learning it from scenes. (**C**) Illustration of the border effect. All conventions are the same as in (**A,B**).

Another important class of saliency effects involves collinear facilitation. We tested the model with simple arrangements of bars that are known to produce higher perceptual salience for collinear (*Col*) than parallel sets of bars (*Par*), relative to a homogeneous texture (*Txt*) of iso-oriented bars (Fig. 12). We used the same length, contrast, and spacing of bars as in Fig. 11. To explain the salience maps, first note that model responses at the center of each bar were always normalized by the outputs of the surround group with the same orientation (i.e. the co-assignment probability was large at every location, due to partial encroachment of the center bar on surround RFs and vice versa); therefore the differences between *Col* and *Par* could not be attributed to different assignments as in Fig. 9, but rather to the asymmetry of the covariance matrices as explained below.

First, we consider the bars on the boundary regions. In Fig. 12A, the boundary includes the middle row of collinear horizontal bars (*Col*), and the neighboring rows of parallel vertical bars above and below (*Par*). For such bars, only a portion of the surround has the same orientation as the center, leading to overall less surround activation at the boundary than inside the homogenous regions (*Txt*; i.e., in Fig. 12A, where each vertical bar is entirely surrounded by vertical bars). Since the co-assignment probability was large for both the border and homogenous region, we observed weaker surround suppression, and thus larger salience, at the border than in the homogenous regions.

In addition, we observed a larger salience enhancement for the middle row of bars, relative to the homogeneous region, when they were collinear (Fig. 12A) than parallel (Fig. 12B). Collinear and parallel salience enhancement were defined as *(Col-Txt)/Txt* and *(Par-Txt)/Txt*, respectively. The larger collinear enhancement was due only in part to the stronger RF linear outputs for collinear stimuli (since surround bars encroached on the center RF) and was largely attributable to the covariance matrices learned from scenes. As explained in Materials and Methods, in the co-assigned model components, the collinear surround RFs had higher variance, and higher covariance with the center RF, than the parallel RFs. This led to smaller normalization signals, and therefore weaker suppression, for collinear than parallel stimuli. As a control, we computed the salience maps with a reduced version of the model that assumed diagonal covariance matrices and equal variances, and found that the difference of salience enhancement between *Col* and *Par* was reduced for Fig. 12A, while it was increased for Fig. 12B, relative to the full model. Therefore, the control was less compatible with collinear facilitation.

The two examples above are to some extent combined in the border effect in stimuli such as Fig. 12C, where the side of the border on which the set of bars adjacent to and defining the border are collinear is perceptually more salient than the other [75]. The salience map computed by the model also showed this effect, due to the covariance structure, as explained above.

## Discussion

We have addressed spatial contextual influences in early vision by introducing a statistical model that is rich enough to capture the coordination that exists amongst spatially distributed V1-like RFs in natural scenes. Our model took explicit account of the spatial heterogeneity of scenes. Inference in the model amounted to a generalized form of divisive normalization from each RF's surround. The model reproduced a host of V1 neural surround nonlinearities, including both suppression and facilitation, such as RF expansion at low contrast (Fig. 7); a number of phenomena relating to orientation tuning (Fig. 8); and spatial asymmetry (Fig. 9,10) of surround modulation. Combining model responses across orientations yielded perceptual salience phenomena of pop-out, enhancement of collinear borders and texture border assignment (Fig. 11,12). Thus, we have shown that a whole range of qualitative behaviors can be captured by a model that is based on encoding the statistical properties of natural images. This provides a principled account of contextual phenomena which makes specific predictions (such as Fig. 9B–D) that are straightforward to test using existing methods and techniques. It can also explain contextual effects well beyond the examples we provided.

Many important mechanistic accounts of contextual interactions have included ideas about excitatory and inhibitory connections and their links to divisive normalization, e.g. [42], [78]–[80], and surround effects, e.g. [59], [63]. Our model is not a competitor to these, but rather is complementary, adding a layer of interpretation that indicates the nature of the information processing implemented by mechanistic approaches. Specifically, we suggested that contextual modulation is sensitive to the statistical homogeneity of the inputs. Clarifying the underlying mechanisms is an issue that deserves further exploration, and might involve the different firing thresholds or diversity across types of interneurons [59], [60], or may be a property of the cortical network involving input-dependent state switching (e.g. [61]), changes in functional connectivity [62], or the exact balance between excitatory and inhibitory conductances [63].

In addition, surround modulation was more often engaged when stimulating collinear than parallel regions of the surround; but, when surround modulation was engaged, collinear responses were enhanced relative to parallel. These behaviors, due to the form of the covariances learned from natural scenes, may also find a basis in the known specificity and anisotropy of horizontal (e.g. [52]–[55]) and feedback connections (e.g. [56]–[58]) that are thought to mediate V1 contextual interactions. It is important to note also that, differently from most of the mechanistic models mentioned above, our model in its current form does not include free parameters that can be fit to specific experimental datasets; the parameters are instead learned from natural scenes, in a process analogous to development, and then fixed.

### Stimulus-dependent surround normalization

Two main statistical innovations were critical for capturing the full set of biological data. First, our model provides a formal treatment of the idea of statistical homogeneity vs heterogeneity of visual inputs. We proposed that V1 is sensitive to whether center and surround responses to a given stimulus are inferred to be dependent or independent based on the statistical structure of natural scenes. This sensitivity is actually required to capture the stronger statistical dependence apparent within spatially extended visual objects. In our model, it implies that surround modulation is fully engaged by stimuli comprising extended single objects, but disengaged by stimuli with entirely independent center and surround.

This (dis)engagement proved to be crucial to obtain the neural effects in Fig. 7–9. In particular, the model, by choosing among multiple normalization pools with different tuning characteristics, captured the observation that the most suppressive surround orientation is the one matched to the center stimulus, not the center preference (Fig. 8C), which had not previously been modeled. Cavanaugh et al. [9] attributed the effect partly to the encroachment of the surround annulus on the center RF, a factor that they reduced, but could not eliminate entirely, by introducing a gap in the stimuli. In the model we had fuller control over the RFs and the encroachment for the stimuli of Fig. 8A,C, suggesting that at least in part the effect might reflect genuine surround modulation, and can be explained on a statistical basis. The inclusion of multiple surround pools also allowed our model to capture the narrowing of orientation tuning curves with large stimuli (Fig. 8E; see also [35], [81]). The assignments inferred in Fig. 8A are also consistent with (and thus provide statistical support for) those assumed by fiat in the idealized model of [70], where they were important for capturing repulsive and attractive perceptual biases in the tilt illusion.

The flexibility of the normalization pool also implied that the model could exhibit surround facilitation. While a strong (and co-assigned) surround was suppressive, a weak (and co-assigned) surround was facilitative, when compared to responses in the absence of surround (not co-assigned). The magnitude of the modulation was larger for weaker center RFs outputs (Fig. 6). We suggest that this feature of the model can unify disparate experimental observations that weak surround stimulation (e.g. using large gaps, or small collinear stimuli) in conjunction with weak center stimulation (e.g. low contrast) can lead to facilitation [10], [12], [73], [74]. However, the precise dependence of surround tuning, and facilitation, on the optimality of the center stimulus has not been explored systematically, and further experiments will be required to test this general prediction of the model. We should note also that facilitation of an optimal center stimulus by a surround stimulus with largely different or orthogonal orientation, which has also been observed experimentally [9], [10], [67], could be explained more simply by dis-inhibition (or inhibition of the suppressive surround, [9], [35]). Such an explanation demands some form of recurrent processing that is missing in our current model.

### The impact of linear correlations

The second key statistical feature in our model, namely the RFs covariance matrices, reflects the structure of linear correlations found in natural images. For image patches that the model determined to be homogenous, we observed larger variance for the central RF and its collinear neighbors, and larger covariance between the collinear neighbors, than for any other surround location (Fig. 4). This impacted divisive normalization in two important ways that have not been explored previously, and that allowed us to address both the spatial asymmetry of cortical surround modulation, and collinear enhancement of salience with simple displays.

At high contrast, collinear surrounds suppress targets more than parallel surrounds. The model reproduced this characteristic (Fig. 9A) because collinear stimuli are more likely to engage surround modulation. In addition, it predicted that whether collinear grating patches suppress or facilitate a target depends on stimulus contrast and angular size (Fig. 9B), consistent with the limited data on collinear facilitation for unmatched contrasts (low in the center, high in the surround [73]). The latter crucially depended on the larger probability for a high than low contrast central stimulus to engage surround normalization by itself, a fact that also led to “far” surround facilitation [12]. Also, by learning the covariance structure individually for different natural images (Fig. 10), we suggested a possible relation between the scatter of positional biases observed experimentally with both iso- and orthogonally oriented surround patches, and scene statistics.

Furthermore, the model generated salience maps that exhibited collinear enhancement and texture border assignment with simple bar displays (Fig. 12). These stimuli always engaged surround modulation in the model. Due to the correlation structure of Fig. 4, for any bar in the display, the collinear neighbors contributed less to the normalization signal, and so were less suppressive, than the parallel neighbors (Materials and Methods). This might also provide a basis for contour integration, namely the pop-out of a contour composed by collinear, but spatially separated, oriented bars surrounded by randomly oriented distractors. Excitatory interactions among collinear RFs led to contour integration in a dynamical model of V1 saliency [75] and in other computational approaches [82]–[85]. In addition, Li et al. [86] reported that the firing rates of V1 neurons in monkey reflect contour integration only after the animals learned to perform contour detection. Our model produced only marginal contour enhancement with complex bar stimuli. However, since we derived collinear interactions from scene statistics, it is possible that supervised training on contours may further amplify such interactions. Alternatively, our model may need to include higher order statistics [87]–[89] that could be learned at higher processing stages [90].

### Relationship to other models

Our statistical modeling relates most closely to a number of recent powerful approaches in computer vision and statistical learning which have not been directly applied to contextual effects in neuroscience. These include hierarchical models for unsupervised learning of statistical structure in images (e.g. [91]), extensions of Independent Component Analysis (e.g. [31], [92]), and models that explain image patches based on the competition, rather than linear superposition, of independent components (e.g. [93]). Other variants of the GSM have proposed schemes to learn RF covariance matrices with applications to computer-vision problems such as image denoising and quality assessment (e.g. [94], [95]); in particular, Hammond and Simoncelli, 2008 [96] used a mixture of GSMs that allowed for mixing based on an orientedness prior and allowed them to improve noise removal in local oriented structure.

The model of Schwartz et al. [37] allowed for arbitrary pooling of RFs under multiple mixers. This started from a bank of linear RFs over the full visual field, and a collection of normalization pools. Each RF learned a prior affiliation with a number of the pools across a whole collection of image patches; but for any single patch, could only be normalized by a single pool. This resulted in an arrangement in which pools varied in shape and composition thus covering a region of visual space with some probabilistic overlap (see Fig. 11 of [37]). This, and other related scene statistics models [36], perhaps suggest some analogy of RF arrangements to what has been found in the visual periphery (e.g. [97]). However, the models in [36], [37] were not examined from the perspective of biological data. In order to address cortical data, our current approach explored the simplest alternative which still permitted formal separation between center and surround: this required an approximation in which multiple surround RFs can coordinate with a center RF as a group, but not individually. This simplification was crucial to make the covariance optimization computationally tractable, but in the simulation results it was counterbalanced by the fact that the covariance allowed for different weightings of the surround (e.g. Fig. 9,10 where the collinear and parallel regions of the surround receive different assignments). However, for more complex stimuli than those considered in this paper, it remains to be seen how well the approximation works in capturing neural outputs; furthermore, relaxing the approximation will be necessary when considering e.g. simultaneous responses of populations of neurons. It will be important in future work to explore models of intermediate complexity that would still allow flexibility in the surround assignments.

Certain other statistically-minded approaches to computer vision have addressed the implications for biological vision. Schwartz and Simoncelli [30] linked surround divisive normalization to scene statistics by learning divisive weights; however they did not consider linear correlations, nor the concept of stimulus-dependent normalization pools. Karklin and Lewicki [34] also proposed that neural responses encode local statistical variations in the inputs. This led to an impressive set of complex-cell non-linearities, though not explicitly related to divisive normalization, nor covering as wide a range of surround effects. Other computational paradigms of predictive coding [98] modeled divisive surround modulation in depth [35], but did not address spatial non-homogeneities. Some models (e.g. [15], [16]) explained a large range of perceptual salience effects based on cortical-like center-surround interactions, but these have not reproduced V1 surround modulation in detail. Furthermore, by contrast with the dynamical modeling [14], [19]–[21], [75], [76], none of the above accounts tied together neural and perceptual salience effects.

### Future directions

A natural extension of our approach would be to optimize the sampling of visual features and test how well the model predicts V1 responses to complex scenes; and their effects on perceptually assessable factors such as salience detection under controlled manipulation of the assignments, and gaze patterns on natural images [15]. Recurrent implementations of V1 saliency [75] captured also subtle perceptual phenomena, such as the medial axis effect; these elude our model, and might require more cooperative interactions among the units. More generally, context is pervasive in time as well as space [99]: extending the model to learn statistical regularities in natural movies could provide an underpinning for the temporal characteristics of V1 responses and perceptual reports, such as adaptation and its dual in the form of surprise responses [100]. It remains an open question as to whether these contextual effects collectively represent the zenith of V1 processing, and whether and how the same computational principles apply to V2 and beyond.

## Supporting Information

Figure S1 The natural images used to train the model.
(TIF)

Figure S2 Orientation tuning of the center is contrast-invariant. Model tuning curves at different contrast are approximately scaled versions of each other, a characteristic feature of V1 tuning. This holds across different stimulus sizes, both smaller and larger than the receptive field size.
(EPS)

Figure S3 Perceptual pop-out in the model depends on the bars length, separation, and contrast. The red box correspond to the configuration used in Fig. 10, main text.
(TIF)

Text S1 Equations and examples of the linear filters used in the simulations.
(PDF)

Text S2 Mathematical details on the training algorithm.
(PDF)

Text S3 Extended simulation results on surround orientation tuning as a function of contrast, and center and surround stimulus size.
(PDF)

Text S4 Additional simulation results on the positional bias. Cardinal axis effects and variability across different natural images can produce a scatter of positional biases consistent with that observed in V1 populations.
(PDF)
