# Supplementary figures and images for: Cortical Surround Interactions and Perceptual Salience via Natural Scene Statistics
Source: PLoS Comput Biol. 2012 Mar 1;8(3):e1002405. doi: 10.1371/journal.pcbi.1002405 (PMC3291533; doi:10.1371/journal.pcbi.1002405)

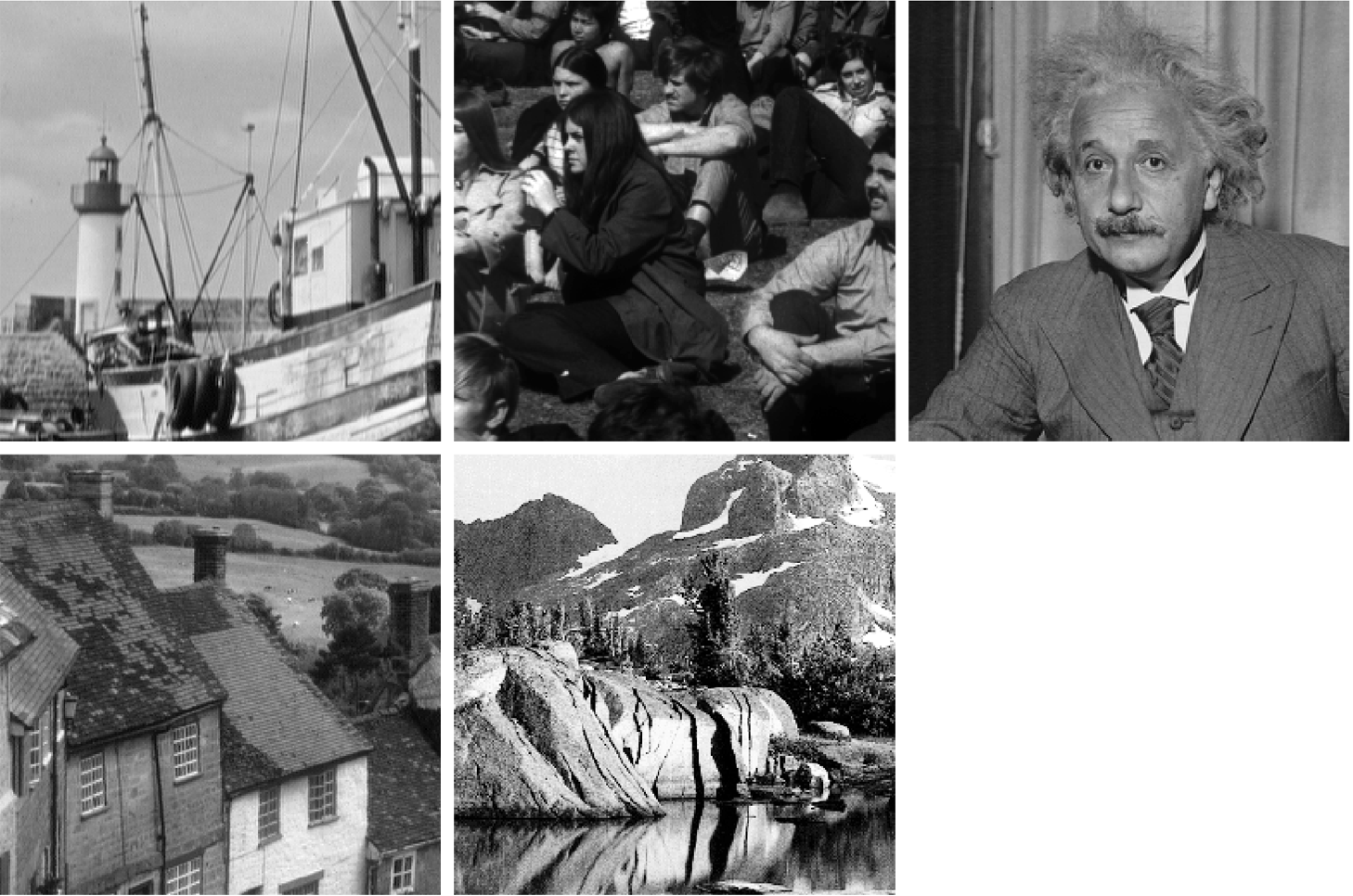

Supplement: Figure S1 — The natural images used to train the model. (TIF) [file pcbi.1002405.s001.tif]

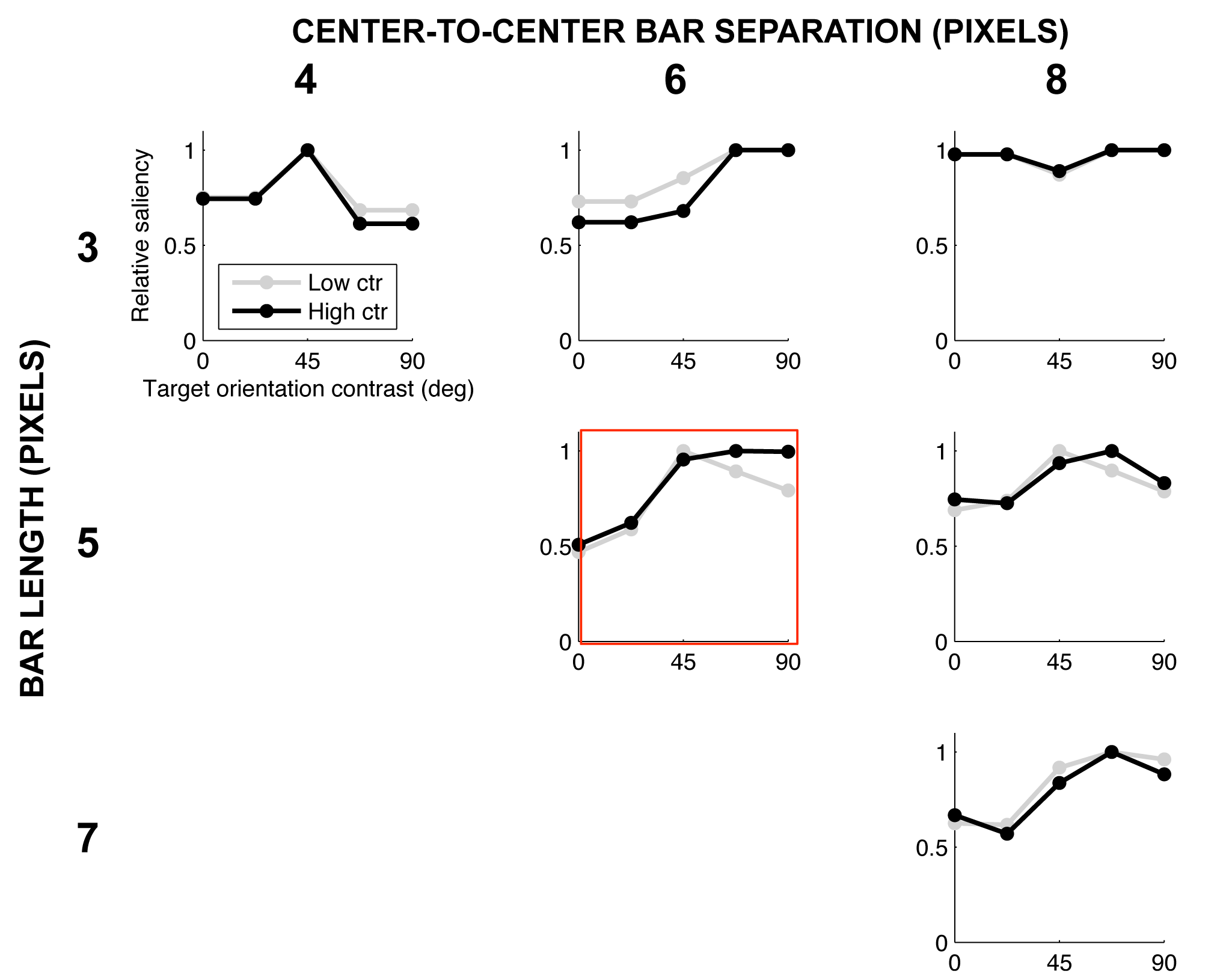

Supplement: Figure S3 — Perceptual pop-out in the model depends on the bars length, separation, and contrast. The red box correspond to the configuration used in Fig. 10, main text. (TIF) [file pcbi.1002405.s003.tif]
