# Supplementary material for: Cortical Surround Interactions and Perceptual Salience via Natural Scene Statistics
Source: PLoS Comput Biol. 2012 Mar 1;8(3):e1002405. doi: 10.1371/journal.pcbi.1002405 (PMC3291533; doi:10.1371/journal.pcbi.1002405)
Supplement: Text S1 — Equations and examples of the linear filters used in the simulations. (PDF) [file pcbi.1002405.s004.pdf]

TEXT S1: LINEAR FILTERS

Linear filters are derived from the first level of a steerable pyramid [1], extended to include complex analytic filters, that is, the real and imaginary parts correspond to a pair of even- and odd-symmetric filters [2]. The filters used in this transformation are polar-separable in the Fourier domain, where they may be written as:

$$L(r,\phi) = \begin{cases} 2\cos(\frac{\pi}{2}\log_2\frac{4r}{\pi}), & \frac{\pi}{4} < r < \frac{\pi}{2} \\ 2 & r \leq \frac{\pi}{4} \\ 0 & r \geq \frac{\pi}{2} \end{cases}$$

$$B_j(r,\phi) = H(r)G_j(\phi), \quad j \in [0, J-1],$$

with radial and angular parts

$$H(r) = \begin{cases} \cos(\frac{\pi}{2}\log_2\frac{2r}{\pi}), & \frac{\pi}{4} < r < \frac{\pi}{2} \\ 1 & r \geq \frac{\pi}{2} \\ 0 & r \leq \frac{\pi}{4} \end{cases}$$

$$G_j(\phi) = \begin{cases} \alpha_J \left[\cos\left(\phi - \frac{\pi_j}{J}\right)\right]^{J-1}, & \left|\phi - \frac{\pi_j}{J}\right| < \frac{\pi}{2} \\ 0 & \text{otherwise}, \end{cases}$$

where J is the number of spatial scales indexed by j, an r, ϕ are polar frequency coordinates, and $\alpha_J = 2^{J-1}\frac{(J-1)!}{\sqrt{J[2(J-1)]!}}$.
Two example filters are shown in fig. 1

References

[1] E.P. Simoncelli, W.T. Freeman, E.H. Adelson, and D.J. Heeger. Shiftable multi-scale transforms. *IEEE Trans. Info. Theory*, 38:587–607, 1992.

[2] J. Portilla and E. P. Simoncelli. A parametric texture model based on joint statistics of complexwavelet coefficients. *International Journal of Computer Vision*, 40(1):49–71, 2000.

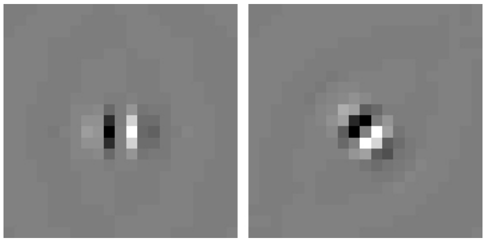

Figure 1. Two examples of the linear filters used in the simulations [1, 2].
