# Supplementary material for: Cortical Surround Interactions and Perceptual Salience via Natural Scene Statistics
Source: PLoS Comput Biol. 2012 Mar 1;8(3):e1002405. doi: 10.1371/journal.pcbi.1002405 (PMC3291533; doi:10.1371/journal.pcbi.1002405)
Supplement: Text S2 — Mathematical details on the training algorithm. (PDF) [file pcbi.1002405.s005.pdf]

TEXT S2: MODEL TRAINING VIA GENERALIZED EM

Before discussing the training algorithm, we briefly recall the notation introduced in the main text. We considered a mixture of five GSMs, defined on five groups of variables that describe the responses of oriented linear filters to natural images: one center group and four surround groups. The center group comprises filters with orientations 0, 45, 90 and 135 degrees from vertical, all centered at the same spatial location; each surround group comprises filters with equal orientation (corresponding to one of the center orientations), located on a circle surrounding the center filters with radius 6 pixels (see Figure 3, main text). Associated with each group is an independent, positive mixer variable ν for which we assume Rayleigh distribution (Equation 3, main text). Associated with each linear filter are a zero–mean Gaussian variable, and a GSM variable defined as the product of the Gaussian times the appropriate mixer. We will denote by $\boldsymbol{\kappa}$ a n_k-dimensional vector of Gaussian variables for the center, where n_k corresponds to the number of orientations (i.e., 4) used for the center filters; and by $\boldsymbol{\Sigma}$ a matrix of Gaussian variables for the surrounds, where each of the n_k columns corresponds to a given orientation of the surround filters, and each of the n_S rows to a given position of the surround filters (i.e., $n_S = 8$). Similarly, the GSM variables describing the responses of center filters are denoted by $\mathbf{k}$, and those describing surround filters are denoted by the matrix $\mathbf{S}$. We then considered five *assignment* configurations that define the structure of dependencies in each of the mixture components. The first four, ξ_θ for $\theta \in \Delta = \{0, 45, 90, 135\}$, comprise the cases where the center group and the surround with orientation θ are *co–assigned* to a common mixer; the fifth assignment configuration, $\xi_\star$, is the case where target and context groups are all mutually independent, and therefore the Gaussians associated with each group are multiplied by their own independent mixers. In the main text (Equation 4) we derived the joint distribution of the center and surround RF variables under ξ_θ, which we rewrite also here for convenience:
(S1)

$$p(\mathbf{k}, \mathbf{S} \,|\, \xi_\phi) = \frac{\det((C^{\phi}_{kS})^{-1})^{\frac{1}{2}}}{(2\pi)^{\frac{n}{2}}} \, \frac{\mathcal{B}(1 - \frac{n}{2}\, ; \, \lambda^{\phi}_{kS})}{(\lambda^{\phi}_{kS})^{(\frac{n}{2}-1)}} \prod_{\theta \neq \phi} \frac{\det((C^{\theta}_{S})^{-1})^{\frac{1}{2}}}{(2\pi)^{\frac{n_S}{2}}} \, \frac{\mathcal{B}(1 - \frac{n_S}{2}\, ; \, \lambda^{\theta}_{S})}{(\lambda^{\theta}_{S})^{(\frac{n_S}{2}-1)}}$$

The parameters to be estimated are the covariance matrices of the Gaussians (denoted by $\Theta \doteq \{C_k, C^0_S, C^{45}_S, C^{90}_S, C^{135}_S, C^0_{kS}, C^{45}_{kS}, C^{90}_{kS}, C^{135}_{kS}\}$) and the prior probabilities of the assignment configurations (denoted by $q^\theta \doteq p(\Xi = \xi_\theta)$, for $\theta \in \Delta$ and $q^\star = 1 - \sum_{\theta \in \Delta} q^\theta$, and collectively by $\rho = \{q^0, q^{45}, q^{90}, q^{135}, q^\star\}$); we use a Generalized Expectation Maximization algorithm, namely Expectation Conditional Maximization [1], where a full EM cycle is divided into several subcycles, each involving a full E-step and a partial M-step performed only on one covariance matrix.

E–step: In the E–step we compute an estimate (Q) of the posterior distribution over the assignment variable, given the observed variables $\mathbf{k}, \mathbf{S}$ and the previous

estimates of the parameters $(\rho^{old}, \Theta^{old})$, via Bayes rule:

$$
\text{(S2)} \qquad \begin{aligned} Q(\xi_\theta) &= p(\xi_\theta \,|\, \mathbf{k}, \mathbf{S}; \Theta^{old}) \\ &= \frac{q^{\theta,old}\, p(\mathbf{k}, \mathbf{S} \,|\, \xi_\theta; \Theta^{old})}{\sum_{\phi\in\Delta} q^{\phi,old}\, p(\mathbf{k}, \mathbf{S} \,|\, \xi_\phi; \Theta^{old}) + q^{\star,old}\, p(\mathbf{k}, \mathbf{S} \,|\, \xi_\star; \Theta^{old})} \end{aligned}
$$

and similarly for $Q(\xi_\star)$.

M–step: In the M–step we maximize the complete–data Log Likelihood, namely:

$$
\text{(S3)} \qquad \begin{aligned} f &= \sum_{\theta\in\Delta} Q(\xi_\theta) \log\left[p(\mathbf{k}, \mathbf{S}, \xi_\theta \,|\, \Theta)\right] + Q(\xi_\star) \log\left[p(\mathbf{k}, \mathbf{S}, \xi_\star \,|\, \Theta)\right] \\ &= \sum_{\theta\in\Delta} Q(\xi_\theta) \log\left[q^\theta\, p(\mathbf{k}, \mathbf{S} \,|\, \xi_\theta; \Theta)\right] + Q(\xi_\star) \log\left[q^\star\, p(\mathbf{k}, \mathbf{S} \,|\, \xi_\star; \Theta)\right] \end{aligned}
$$

where the second line is obtained applying Bayes rule. The gradient of f w.r.t. the parameters comprises four groups of partial derivatives:

$$
\text{(S4)} \qquad \frac{\partial f}{\partial q^\theta} \quad ; \quad \frac{\partial f}{\partial (C^\theta_{kS})^{-1}} \quad ; \quad \frac{\partial f}{\partial (C^\theta_{S})^{-1}} \quad ; \quad \frac{\partial f}{\partial (C_k)^{-1}}
$$

Setting to zero the first term, we obtain the analytical solution for $\hat{\rho} \doteq \arg\max_\rho [f]$ after some simple algebra:

$$
\text{(S5)} \qquad q^\theta = Q(\xi_\theta)
$$

The zeros of the other terms cannot be solved analytically, and we solved for the optimal covariance matrices using conjugate gradient descent. The explicit expression for the second term of the gradient is obtained as follows:

$$
\text{(S6)} \qquad \frac{\partial f}{\partial (C^\theta_{kS})^{-1}} = Q(\xi_\theta) \frac{\frac{\partial p(\mathbf{z} \,|\, \xi_\theta; \Theta)}{\partial (C^\theta_{kS})^{-1}}}{p(\mathbf{z} \,|\, \xi_\theta; \Theta)}
$$

where, for better readability of the following equations, we denoted by $\mathbf{z}$ the vector composed by the GSM variables associated with the center and the surround with orientation θ. First, the numerator is:

(S7)

$$
\begin{aligned} \frac{\partial p(\mathbf{z} \,|\, \xi_\theta; \Theta)}{\partial (C^\theta_{kS})^{-1}} &= \frac{\partial}{\partial (C^\theta_{kS})^{-1}} \int d\nu \frac{\nu^{1-n}}{(2\pi)^{\frac{n}{2}} \det(C^\theta_{kS})^{\frac{1}{2}}} \exp\Big\{ -\frac{\nu^2}{2} - \frac{\mathbf{z}^\top (C^\theta_{kS})^{-1} \mathbf{z}}{2\nu^2} \Big\} \\ &= \int d\nu \frac{\nu^{1-n} \det((C^\theta_{kS})^{-1})^{\frac{1}{2}}}{(2\pi)^{\frac{n}{2}}} \Big(-\frac{\mathbf{z}\mathbf{z}^\top}{2\nu^2} \Big) \exp\Big\{ -\frac{\nu^2}{2} - \frac{\mathbf{z}^\top (C^\theta_{kS})^{-1} \mathbf{z}}{2\nu^2} \Big\} \\ &+ \int d\nu \frac{\nu^{1-n} \det((C^\theta_{kS})^{-1})^{\frac{1}{2}}}{(2\pi)^{\frac{n}{2}}} \Big(-\frac{\det((C^\theta_{kS})^{-1})(C^\theta_{kS})}{2} \Big) \exp\Big\{ -\frac{\nu^2}{2} - \frac{\mathbf{z}^\top (C^\theta_{kS})^{-1} \mathbf{z}}{2\nu^2} \Big\} \\ &= -\frac{\det((C^\theta_{kS})^{-1})^{\frac{1}{2}}}{2(2\pi)^{\frac{n}{2}}} \frac{\mathcal{B}(-\frac{n}{2}\,;\, \lambda^\theta_{kS})}{(\lambda^\theta_{kS})^{\frac{n}{2}}} \mathbf{z}\mathbf{z}^\top + p(\mathbf{z} \,|\, \xi_\theta; \Theta) \frac{(C^\theta_{kS})}{2} \end{aligned}
$$

where the third and fourth lines are obtained integrating by parts and taking the matrix derivatives, and the last line solving the indeterminate integrals. Eventually, using equation (S1), the derivative is given by:

$$
\text{(S8)} \qquad \frac{\partial f}{\partial (C^\theta_{kS})^{-1}} = Q(\xi_\theta) \Big(\frac{(C^\theta_{kS})}{2} - \frac{1}{2\lambda^\theta_{kS}} \frac{\mathcal{B}(-\frac{n}{2}\,;\, \lambda^\theta_{kS})}{\mathcal{B}(1-\frac{n}{2}\,;\, \lambda^\theta_{kS})} \mathbf{z}\mathbf{z}^\top \Big)
$$

The other components of the gradient are obtained in a similar way, and amount to:

$$\frac{\partial f}{\partial (C_S^\theta)^{-1}} = \Big(\sum_{\phi\neq\theta} Q(\xi_\phi)\Big)\Big(\frac{C_S^\theta}{2} - \frac{1}{2\lambda_S^\theta}\frac{\mathcal{B}(-\frac{n_S}{2}\,;\,\lambda_S^\theta)}{\mathcal{B}(1-\frac{n_S}{2}\,;\,\lambda_S^\theta)}\mathbf{z}\mathbf{z}^\top\Big) \tag{S9}$$

where in this case $\mathbf{z}$ denotes only the surround filters with orientation θ; and

$$\frac{\partial f}{\partial C_k^{-1}} = Q(\xi_\star)\Big(\frac{C_k}{2} - \frac{1}{2\lambda_k}\frac{\mathcal{B}(-\frac{n_k}{2}\,;\,\lambda_k)}{\mathcal{B}(1-\frac{n_k}{2}\,;\,\lambda_k)}\mathbf{k}\mathbf{k}^\top\Big) \tag{S10}$$

To evaluate the training results, fig. 1 shows that after training the model the distribution of the estimates of a Gaussian component (see Equation 10 of the main text) is close to an ideal Gaussian with equal variance. In addition, the variance dependency of a pair of center–surround filters - i.e. the bowtie shape in panel b - is eliminated in the joint conditional distribution of the corresponding Gaussian components (panel c).

References

[1] X.L. Meng and D.B. Rubin. Maximum likelihood estimation via the ecm algorithm: A general framework. *Biometrika*, 80(2):267–278, 1993.

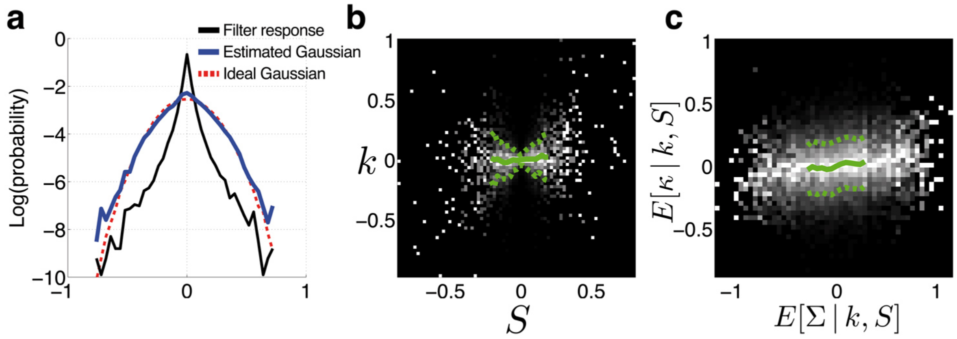

FIGURE 1. Evaluation of the parameters learned from natural scenes. (**a**) Marginal histograms of: (black) the responses of the central vertical RF (see main text, fig. 3) to natural images; (blue) the expected values of the corresponding Gaussian component; and (red) an ideal Gaussian with equal variance. (**b**) Conditional histograms of the outputs of two linear RFs (the vertical central RF, x_c, and a vertical RF from the surround, x_s) in response to natural images. (**c**) Conditional histograms of the expected values of the corresponding Gaussian components. Gaussian estimates are computed according to equation 10 (see main text), with the parameters learned from a database of natural scenes. In (**b,c**) pixel intensity is proportional to probability, with larger values corresponding to brighter pixels; each column is independently rescaled to fill the range of intensities. Solid and dashed lines denote conditional mean and standard deviation respectively.
