# Supplementary material for: Cortical Surround Interactions and Perceptual Salience via Natural Scene Statistics
Source: PLoS Comput Biol. 2012 Mar 1;8(3):e1002405. doi: 10.1371/journal.pcbi.1002405 (PMC3291533; doi:10.1371/journal.pcbi.1002405)
Supplement: Text S3 — Extended simulation results on surround orientation tuning as a function of contrast, and center and surround stimulus size. (PDF) [file pcbi.1002405.s006.pdf]

TEXT S3: SURROUND ORIENTATION TUNING

Figure 8a in the main text show that our model can exhibit suppression and facilitation as a function of orientation difference between a circular grating presented in the center and an annular grating in the surround. The balance of suppression to facilitation in the model depended on the contrast of the stimulus, and on the extent of annular separation between center and surround stimuli, as demonstrated in Fig. 1. Qualitatively similar patterns were reported in [1], suggesting that larger gaps generally result in weaker iso-oriented suppression, whereas, with smaller gaps, the inhibitory mechanisms can mask the facilitation at large orientation differences. In the model, facilitation was virtually absent when the center grating patch extended beyond the central RF.

The simulation results of Fig. 1 however partly fail to correctly reproduce two effects. First, [2] reported that an iso–oriented surround stimulus is always suppressive, regardless of the contrast of the center and surround stimulus; in our simulations this was the case for all the conditions with a small gap between center and surround, but we instead found facilitation at low contrast for large gap sizes (11, 13 pixels) combined with center patches smaller than the center filter (5, 7 pixels). This was due to the fact that, given the coarse sampling of the surround in the model, stimuli with a large gap size drive the surround linear filters only weakly.

Second, [3] found that, for a fixed high contrast surround stimulus, a reduction in the contrast of the center stimulus produces a larger increase in suppression for orthogonal than iso–oriented surrounds (Fig. 2a). We tested our model under the same conditions, and found that the model reproduces this effect when the center stimulus is at least as large as the center filter (9, 11 pixels) but generally fails to do so for smaller center sizes (Fig. 2b; note that the red points in this figure correspond to unmatched center–surround contrasts, thus differ from the low contrast curves of Fig. 1).

References

[1] H. E. Jones, W. Wang, and A. M. Sillito. Spatial organization and magnitude of orientation contrast interactions in primate v1. *J Neurophysiol*, 82:2796–2808, 2002.

[2] J. R. Cavanaugh, W. Bair, and J. A. Movshon. Nature and interaction of signals from the receptive field center and surround in macaque v1 neurons. *J Neurophysiol*, 88(5):2530–2546, 2002.

[3] J. R. Cavanaugh, W. Bair, and J. A. Movshon. Selectivity and spatial distribution of signals from the receptive field surround in macaque v1 neurons. *J Neurophysiol*, 88(5):2547–2556, 2002.

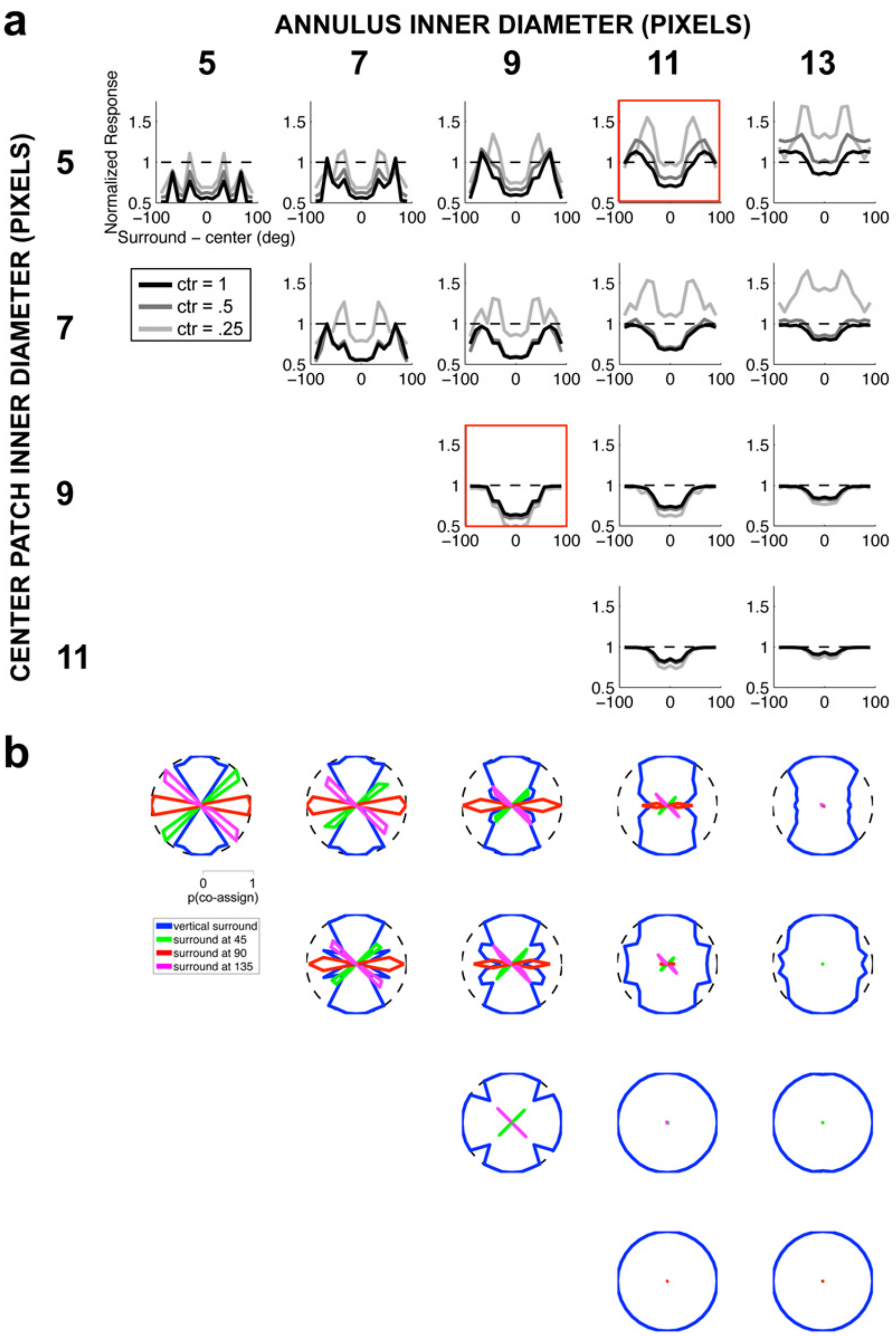

Figure 1. Surround orientation tuning in the model depends on the diameter of the central grating patch, the inner diameter of the annulus, and contrast. (**a**) Model responses; (**b**) co-assignment probabilities for the stimuli with contrast = 0.5. All conventions are the same as in Fig. 8a, main text; the red boxes in (**a**) correspond to the configurations used in Fig. 8a, main text.

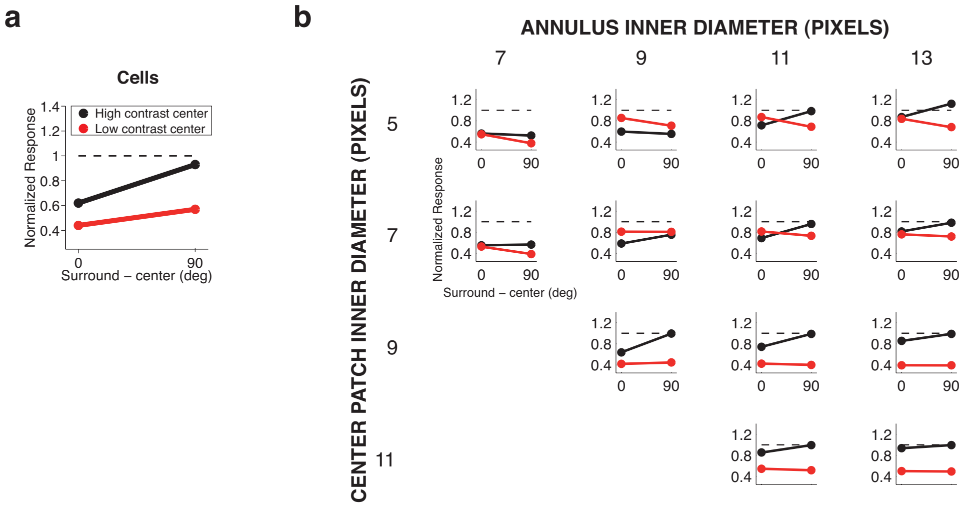

FIGURE 2. Dependence of surround orientation tuning on the center contrast. (**a**) Data adapted from [3]; (**b**) model responses. Black circles represent the surround modulation observed with high contrast stimuli; red circles correspond to the unmatched contrast condition, with the surround fixed at high contrast, the center at low contrast.
