# Supplementary material for: Cortical Surround Interactions and Perceptual Salience via Natural Scene Statistics
Source: PLoS Comput Biol. 2012 Mar 1;8(3):e1002405. doi: 10.1371/journal.pcbi.1002405 (PMC3291533; doi:10.1371/journal.pcbi.1002405)
Supplement: Text S4 — Additional simulation results on the positional bias. Cardinal axis effects and variability across different natural images can produce a scatter of positional biases consistent with that observed in V1 populations. (PDF) [file pcbi.1002405.s007.pdf]

TEXT S4: SURROUND SPATIAL ASYMMETRY

Cavanaugh et al. [1] reported that different locations of the RF surround can have different modulatory effects on neural responses. In Fig. 9 and 10 of the main text we showed that our model can account for the positional bias observed in the data when stimulating the center and surround both with similarly oriented, and orthogonally oriented, grating patches; and that optimizing the model parameters on different natural images produced a variability in such positional bias analogous (although smaller, see the Discussion in the main text) to that seen in the data. Here we provide further simulations.

Figure 1a shows specific examples of the bias with orthogonally oriented surround patches, at different combinations of size and contrast. Figure 1b also illustrates how, in the model, orthogonally oriented surround patches placed at the side of the RF led to higher co–assignment probability for the orthogonal (i.e. horizontal) normalization pool, compared to patches placed at the end of the RF, leading to stronger suppression from the former than the latter. Figure 2 shows the results for the iso–oriented surround patches, with the same contrast and size values used in figure Figure 1, as a reference.

Figure 3 shows the effect of not imposing rotational symmetry of the covariance matrices learned from natural images. Due to the predominance in scenes of oriented structures along the cardinal axes, the positional bias was more pronounced, and contrast–invariant, for the vertical and horizontal model units than for the diagonal units. This suggests that some of the variability of the bias observed in V1 may also reflect a property of the natural visual environment.

In Fig. 10 of the main text we extended the above result by training the model on multiple individual images. Figure 4c,d illustrates the covariance matrices for the unit (red circle in Fig. 4a) that had the largest deviation from the population trend. Such matrices, resulting from optimizing the model on the image shown in Fig. 4b, differ from those in Fig. 4 of the main text in that the variance of the collinear surround filters, and their covariance with the center filter, was slightly weaker than for the parallel filters.

References

[1] J. R. Cavanaugh, W. Bair, and J. A. Movshon. Nature and interaction of signals from the receptive field center and surround in macaque v1 neurons. *J Neurophysiol*, 88(5):2530–2546, 2002.

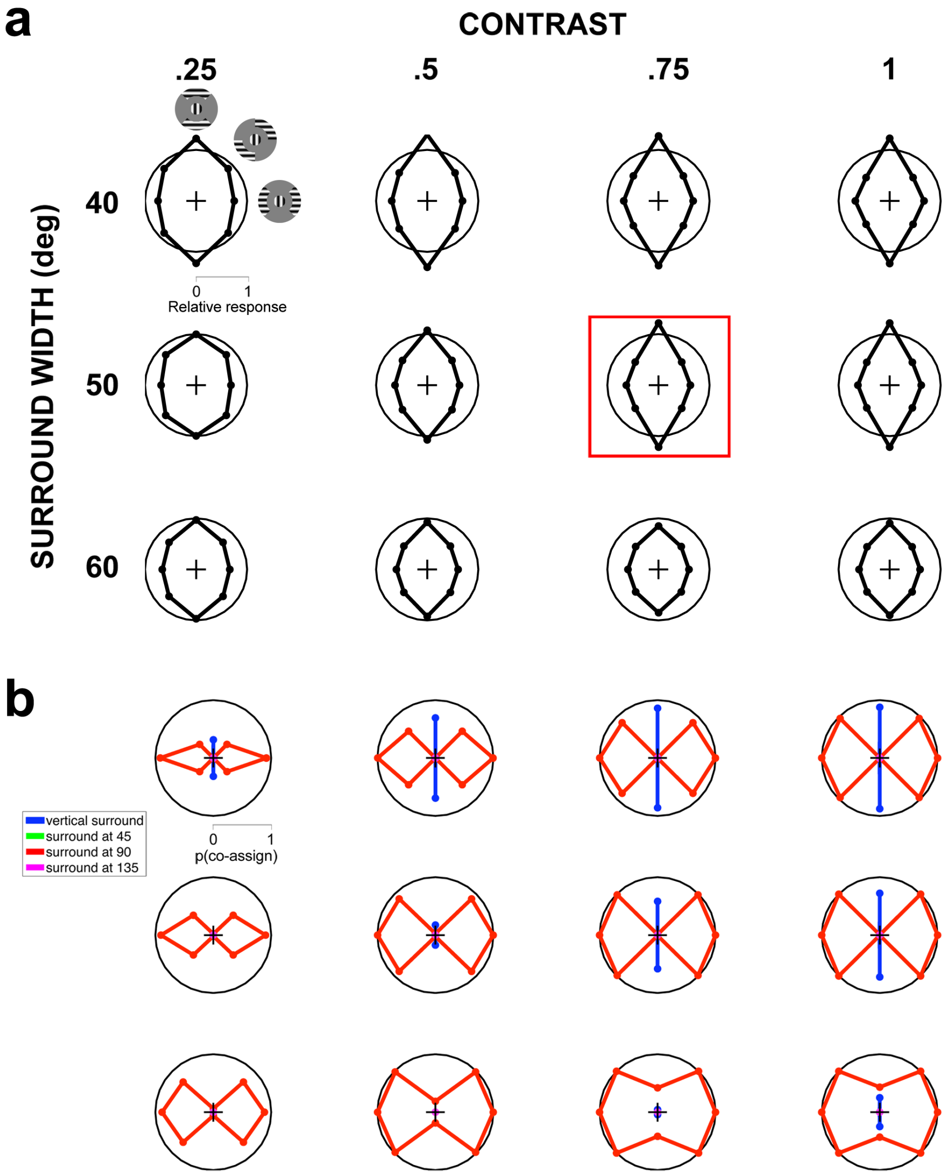

FIGURE 1. Spatial asymmetry of surround modulation with orthogonally oriented surround grating patches. (**a**) Model responses; the stimuli are depicted by the icons in the top-left panel. (**b**) co-assignment probabilities. All conventions are the same as in Fig. 9a, main text; the red box in (**a**) corresponds to the size and contrast used in Fig. 9a, main text (note that Fig.9 used iso-oriented, rather than orthogonal, surround patches).

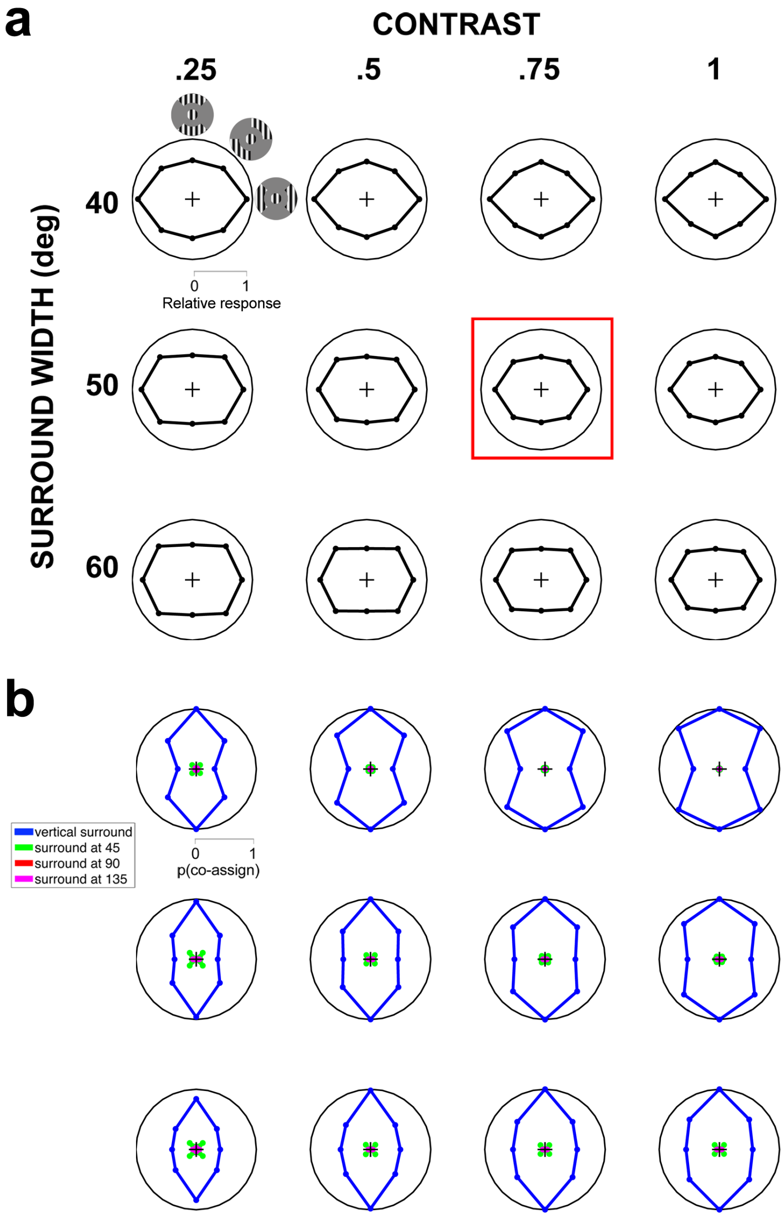

FIGURE 2. Spatial asymmetry of surround modulation with optimally oriented surround grating patches. (**a**) Model responses; the stimuli are depicted by the icons in the top-left panel. (**b**) co-assignment probabilities. All conventions are the same as in Fig. 9a, main text; the red box in (**a**) corresponds to the size and contrast used in Fig. 9a, main text.

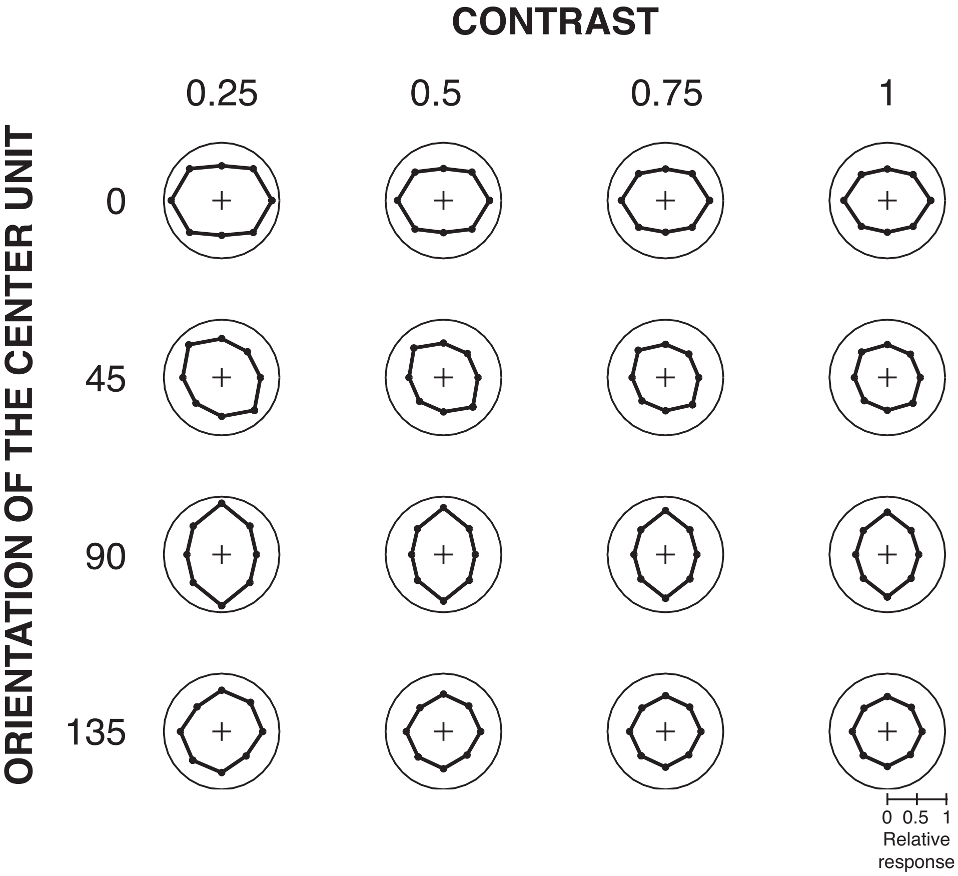

Figure 3. Spatial asymmetry of surround modulation reflects the predominance of cardinal orientations in scenes. (**a**) Examples of model responses as a function of the center unit orientation and stimulus contrast. In each panes, both center and surround patches orientations are matched to the unit orientation. All conventions are the same as in Fig. 9a, main text.

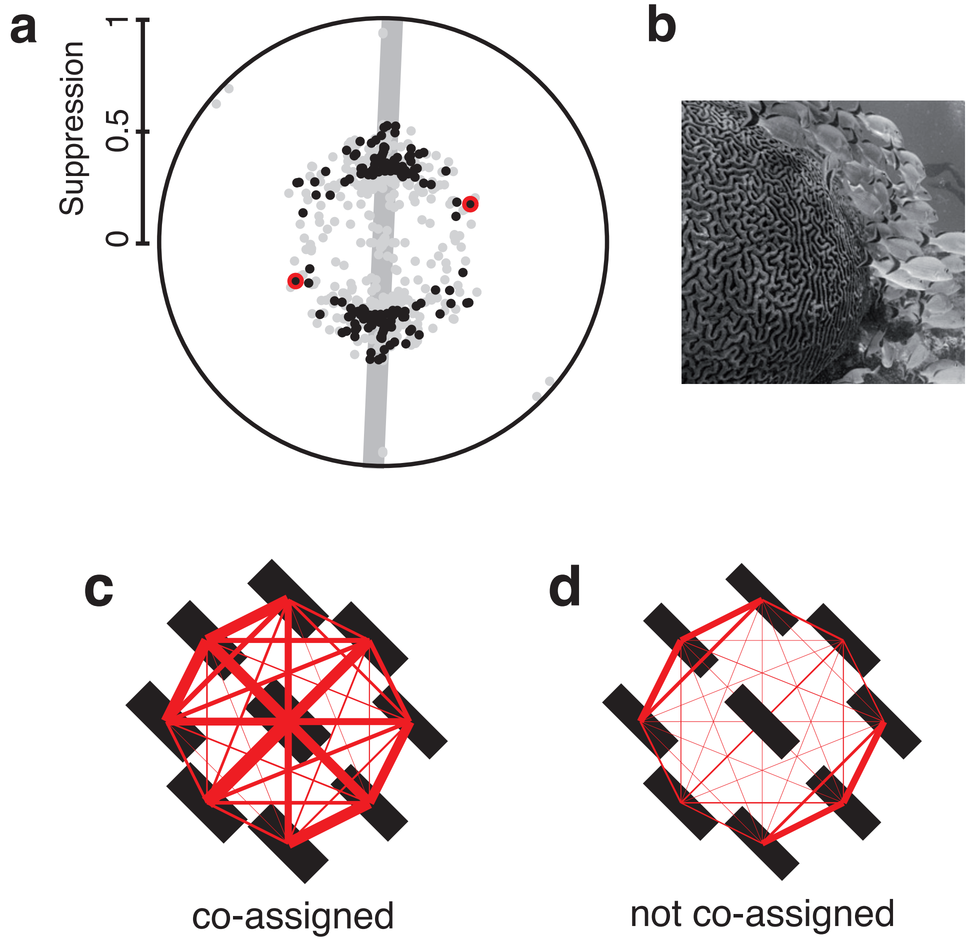

Figure 4. (**a**) The scatterplot is repeated from Fig. 10d main text. The red circle indicates the unit with the largest deviation from the population trend, among those with strong suppression and strong bias. (**b**) The training image and (**c,d**) the corresponding covariance matrices, that produced the surround bias of the red circle in (**a**).
